# Odontogenic exosomes simulating the developmental microenvironment promote complete regeneration of pulp-dentin complex in vivo

**DOI:** 10.1016/j.jare.2024.12.048

**Published:** 2025-01-05

**Authors:** Yifan Wang, Jing Mao, Yujie Wang, Rui Wang, Nan Jiang, Xiaohan Hu, Xin Shi

**Affiliations:** a Center of Stomatology, Tongji Hospital, Tongji Medical College, Huazhong University of Science and Technology, Wuhan 430030, People's Republic of China; b School of Stomatology, Tongji Medical College, Huazhong University of Science and Technology, Wuhan 430030, People's Republic of China; c Hubei Province Key Laboratory of Oral and Maxillofacial Development and Regeneration, Wuhan 430022, People's Republic of China; d Central Laboratory, National Engineering Laboratory for Digital and Material Technology of Stomatology, Beijing Key Laboratory of Digital Stomatology, Peking University School and Hospital of Stomatology, Beijing 100081, People's Republic of China; e Outpatient Department Office, Tongji Hospital, Tongji Medical College, Huazhong University of Science and Technology, Wuhan 430030, People's Republic of China

**Keywords:** Dental pulp stem cells, Exosomes, Odontogenesis, Angiogenesis, Neurogenesis

## Abstract

**Introduction:**

Establishing an optimized regenerative microenvironment for pulp-dentin complex engineering has become increasingly critical. Recently, exosomes have emerged as favorable biomimetic nanotherapeutic tools to simulate the developmental microenvironment and facilitate tissue regeneration.

**Objectives:**

This study aimed to elucidate the multifaceted roles of exosomes from human dental pulp stem cells (DPSCs) that initiated odontogenic differentiation while sustaining mesenchymal stem cell (MSC) characteristics in odontogenesis, angiogenesis, and neurogenesis during pulp-dentin complex regeneration.

**Methods:**

Differential centrifugation was performed to isolate exosomes from normal DPSCs (DPSC-Exos) and DPSCs that initially triggered odontogenic differentiation (DPSC-Od-Exos). The impact of these exosomes on the biological behavior of DPSCs and human umbilical vein endothelial cells (HUVECs) was examined in vitro through CCK-8 assay and Transwell migration assay, as well as assays dedicated to assessing odontogenic, angiogenic, and neurogenic capabilities. In vivo, Matrigel plugs and human tooth root fragments incorporating either DPSC-Exos or DPSC-Od-Exos were subcutaneously transplanted into mouse models. Subsequent histological, immunohistochemical, and immunofluorescent analyses were conducted to determine the regenerative outcomes.

**Results:**

DPSC-Exos and DPSC-Od-Exos revealed no remarkable difference in their characteristics. In vitro analyses indicated that DPSC-Od-Exos significantly facilitated the proliferation, migration, and multilineage differentiation of DPSCs compared with DPSC-Exos. Furthermore, DPSC-Od-Exos elicited a more pronounced effect on the tubular structure formation of HUVECs. Consistently, Matrigel plug assays confirmed that DPSC-Od-Exos exhibited superior performance in promoting endothelial differentiation of DPSCs and stimulating angiogenesis in HUVECs. Notably, DPSC-Od-Exos contributed to complete pulp-dentin complex regeneration in human tooth root fragments, characterized by enriched neurovascular structures and a continuous layer of odontoblast-like cells, which extended cytoplasmic projections into the newly formed dentinal tubules.

**Conclusion:**

By simulating the developmental microenvironment, multifunctional DPSC-Od-Exos demonstrated promising potential for reconstructing dentin-like tissue, vascular networks, and neural architectures, thereby enhancing our understanding of the therapeutic implications of DPSC-Od-Exos in regenerative endodontic treatment.

## Highlights

• Compared with normal exosomes derived from dental pulp stem cells (DPSC-Exos), exosomes secreted by DPSCs undergoing initial odontogenic differentiation (DPSC-Od-Exos) elicited a more robust enhancement on the proliferation and migration as well as odontogenic, endothelial, and neurogenic differentiation of DPSCs.
• DPSC-Od-Exos were superior to DPSC-Exos in prompting the proliferative, migratory, and angiogenic potential of human umbilical vein endothelial cells (HUVECs).
• As reveled by the Matrigel plug assay, DPSC-Od-Exos performed better than DPSC-Exos in ameliorating the formation of vascular tube-like structures of DPSCs and HUVECs.
• After subcutaneous transplantation with DPSCs, DPSC-Od-Exos contributed to the regeneration of complete pulp-dentin complex in human tooth root fragments, which encompassed enriched neurovascular structures and a continuous layer of odontoblast-like cells extending their cytoplasmic projections into the newly formed dentinal tubules.

## Graphical abstract

DPSC-Od-Exos from DPSCs undergoing initial odontogenic differentiation facilitated complete regeneration of pulp-dentin complex by enhancing odontogenesis, angiogenesis, and neurogenesis, shedding light on their promising prospects in advancing regenerative endodontic therapy.

**Figure:**
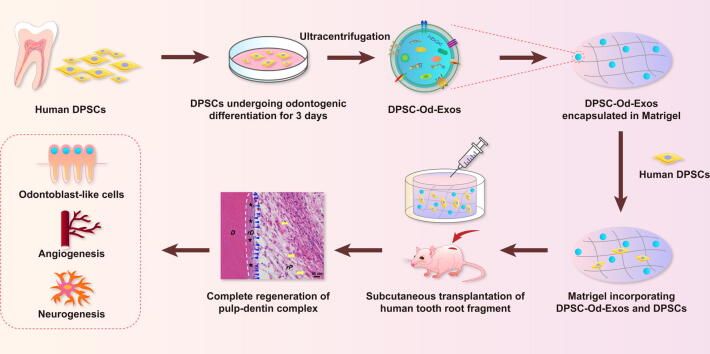

## Introduction

Pulpitis is a prevalent oral inflammatory condition predominantly instigated by bacterial infection. Acute pulpitis is characterized by intense dental pain, which considerably compromises the patient’s oral health and diminishes their overall quality of life. Currently, the preferred clinical therapeutic approach for pulpitis is root canal treatment (RCT), which aims to completely eradicate infected pulp tissue and dentin in the root canal and seal it tightly with biologically inert materials [1], [2]. However, this treatment modality can not be recognized as an ideal strategy. It necessitates the sacrifice of the natural structure and function of pulp-dentin complex and fails to achieve original structural regeneration and functional reconstruction. Consequently, RCT results in the complete loss of tooth vitality, increased tooth brittleness, and enhanced susceptibility to tooth fracture or even tooth loss [3], [4]. It has been declared that following RCT, the risk of missing anterior and premolar teeth escalates by 1.8 times, while the risk associated with molar teeth increases substantially by 7.4 times [5]. In this context, maintaining the structural and functional integrity of pulp-dentin complex plays an indispensable role in preserving tooth vitality and rescuing normal physiological functions of teeth. Regenerative endodontic therapy (RET) has emerged as a promising alternative, focusing on the regeneration of tubular dentin and neurovascular structures within the pulp tissue, which are crucial for recapitulating the nutrient supply, sensory transmission, and immune defense of pulp-dentin complex [6]. As such, RET represents a novel and desirable strategy aimed at the long-lasting, effective, and complete preservation of infected teeth and holds robust potential to substitute traditional RCT. A growing number of animal studies have verified that the RET strategy elicits dramatic superiority over RCT and possesses broad clinical application prospects in inducing pulp-dentin complex regeneration [7], [8], [9]. Nevertheless, it is noteworthy that the clinical translation of RET is confronted with an essential impediment, that is, the reestablishment of an appropriate regenerative microenvironment. Accordingly, there is an urgent need in dental clinics to devise innovative strategies for revitalizing teeth suffering from acute pulpitis and necrosis.

A suitable regenerative microenvironment for pulp-dentin complex engineering will be conducive to a series of critical cellular biological processes, including cell migration, proliferation, odontogenesis, and neurovascular regeneration. Conventional strategies for constructing and maintaining the local microenvironment for pulp-dentin complex regeneration have primarily relied on the combinatorial application of various exogenous growth factors in order to achieve synergistic regulatory effects. Unfortunately, the clinical implementation of these exogenous growth factors poses the risk of tumorigenesis and encounters obstacles such as high cost, short half-life, and complicated combinations. In recent years, advancements in developmental tissue engineering have prompted a paradigm evolution in regenerative strategies. By simulating the developmental microenvironment, these developmentally inspired strategies aim to orchestrate stem cell fate and harness their inherent regenerative capabilities, thereby facilitating the reconstruction of normal functions of injured tissues [8]. To this end, He et al. (2019) illustrated that *Alx3*, a transcription factor pivotal for prenatal tooth development but which regressed during adulthood, when reinstated in DPSCs, remarkably accelerated the regeneration of tubular dentin and vascular-enriched pulp tissue [10]. Moreover, as revealed by Oh et al. (2015), the exogenous replenishment of Cpne7, a preameloblast-secreted diffusible signal responsible for dentinogenesis in developing crowns, played crucial roles in reprogramming nondental-derived MSCs into odontoblast-like cells with cellular processes, which ultimately promoted the regeneration of dentin-like tissue encompassing dentinal tubular structures on the native dentin wall [11]. Taken together, exploiting the developmental events presents enormous promise for discovering optimal signaling molecules to advance regenerative endodontics.

Notably, in addition to critical transcription factors or soluble proteins, exosomes have attracted substantial attention as promising mediators capable of mimicking the developmental microenvironment, thus contributing to the establishment of a beneficial engineering microenvironment in regenerative medicine. Exosomes are nanosized lipid bilayer-enclosed extracellular vesicles (EVs) ranging from 30 to 150 nm in diameter and exhibit a distinct saucer-like morphology. They can be secreted by virtually all cells through the fusion of multivesicular bodies with the cytoplasmic membrane and mediate a diversity of intercellular communications by delivering various biosignaling molecules, such as nucleic acids, proteins, lipids, enzymes, and cytokines [12]. In diverse physiological and pathological settings, exosomes have exerted a extensive regulatory potential. According to Jiang et al. in 2017, under the instruction of exosomes, epithelial and mesenchymal cells in developing tooth organs could reciprocally initiate cellular differentiation and promote extracellular matrix synthesis [13]. Furthermore, Wang et al. (2019) have illuminated that in pathological conditions, exosomes derived from severely inflamed odontoblasts functioned as a unique defensive mechanism, effectively mitigating the apoptosis of adjacent healthy cells [14]. Collectively, the versatile roles of exosomes in tooth organ development and preventative care pave the way for their employment in accelerating tissue engineering. In fact, due to a multitude of advantages, including extensive sources, decreased immunogenicity, inherent targeting ability, favorable safety profile, and proper biofilm-penetrating capacity, exosomes as favorable biomimetic nanotherapeutic tools have become an innovative strategy for remodeling the regenerative microenvironment and promoting the regeneration of various oral tissues, including but not limited to pulp-dentin complex [15], [16], [17], [18]. Aimed at elucidating the capacity of macrophage-derived exosomes to ameliorate the regeneration of pulp-dentin complex, we have recently confirmed through in vitro and in vivo studies that exosomes originating from M2 macrophages (M2-Exos) not only drastically improved the proliferation and migration of DPSCs and HUVECs but also considerably propelled their odontogenic, angiogenic, and neurogenic differentiation competences [19], shedding light on the promising application of M2-Exos in the field of regenerative endodontics. In an attempt to clarify the role of DPSC-Exos in odontogenic differentiation and vascular construction, several research has proposed that DPSC-Exos could markedly augment the odonto/osteogenic potential of DPSCs and support HUVEC proliferation and the formation of capillary-like lumen structures [20], [21], [22], laying a solid foundation for further exploration of their competence in achieving complete regeneration of pulp-dentin complex. Consequently, accumulating efforts are devoted to creating a conducive and inducible microenvironment with exosomes for the advancement of regenerative endodontic practice.

Nonetheless, it is worth pointing out that exosomal function is intimately correlated with their culture microenvironment. Based on the concept of developmental engineering, the specific roles and underlying detailed mechanisms of odontogenic exosomes imitating developmental programs in stimulating pulp-dentin complex regeneration still remain elusive, which warrants further profound understanding. Regarding this, Hu et al. (2019) previously reported that compared with normal DPSC-Exos, DPSC-Od-Exos released by DPSCs at the middle stage of odontogenic differentiation (10 days) elicited a noticeable promoting impact on the expression of key odonto/osteogenic proteins, such as alkaline phosphatase (ALP), dentin matrix protein-1 (DMP-1), and dentin sialoprotein by activating the transforming growth factor β1 (TGF-β1)/Smad2/3 signaling pathway via the delivery of miRNA-27a-5p [23], therefore bringing valuable insights into the establishment of an odontogenesis-related developmental microenvironment. On the other hand, considering that plentiful vascular networks and proper neurogenic recovery represent essential prerequisites during the early stage of pulp regeneration, more intense attention should be paid to investigating the capacity of DPSC-Od-Exos in fostering appropriate vascularization and innervation prior to accomplishing a functional comprehensive regeneration of pulp-dentin complex. Hence, in the present study, we extracted DPSC-Od-Exos, during which DPSCs initiated their odontogenic differentiation while still sustaining MSC stemness to a certain degree, with the goal of examing their multifunctional capabilities in odontogenesis, angiogenesis, and neurogenesis. Encouragingly, we revealed for the first time that DPSC-Od-Exos, enriched with developmental cues, were capable of orchestrating DPSC and HUVEC biological behaviors in vitro and facilitating desirable odontogenesis, vascular reconstruction, and neural recovery in vivo. As a consequence, our findings ascertained that odontogenic exosomes encapsulating MSC and odontogenic characteristics played an imperative role in the reconstitution of an appropriate regenerative microenvironment for pulp-dentin complex and were expected to expedite the clinical translation of regenerative endodontic procedures, demonstrating considerable therapeutic implications and tremendous social benefits.

## Materials and methods

### Animal and ethics

All animal experiments were conducted according to the principles of Laboratory Animal Welfare & Ethics Committee of Tongji Hospital, Tongji Medical College, Huazhong University of Science and Technology (No. TJH-202301004). Impacted wisdom teeth and orthodontic premolars were collected with patients’ informed consent under protocols approved by Medical Ethics Committee of Tongji Hospital, Tongji Medical College, Huazhong University of Science and Technology (No. TJ-IRB202112103).

### Cultivation and identification of human DPSCs

With patients’ informed consent, the clinically extracted premolars or wisdom teeth due to orthodontic treatment were collected and stored in α-modified minimum essential medium (α-MEM) (Gibco, Waltham, MA, USA) supplemented with 100 U/mL penicillin and 100 μg/mL streptomycin (Gibco, Waltham, MA, USA) at low temperatures. To obtain DPSCs, the teeth were repeatedly rinsed with phosphate-buffered saline (PBS) (Gibco, Waltham, MA, USA), then dental pulp tissue was gently cut into small pieces after separating from the crown and the root and subjected to enzymatic digestion with 3 mg/mL collagenase type I (BioFroxx, Einhausen, Germany) and 4 mg/mL dispase (BioFroxx, Einhausen, Germany) for 40 min at 37 °C in a water bath. Subsequently, the cells recognized as primary DPSCs were harvested by centrifugation at 1500 rpm for 10 min and resuspended in sterile growth medium consisting of α-MEM (Gibco, Waltham, MA, USA) and 10% fetal bovine serum (FBS) (Keyi, Wuhan, China). Afterward, the cell suspension was mixed entirely by careful rocking, seeded in T25 cell culture flasks, and cultured at 37 °C in a humidified incubator containing 5% CO_2_.

To detect the osteogenic and adipogenic potential of DPSCs, the primary cells were cultured until passage 6–7 and seeded in 6-well plates at a density of 2×10^5^ cells/well. When the cells reached 70%-80% confluence, 2 mL of osteogenic induction medium (OIM) or adipogenic induction medium (Cyagen, Guangzhou, China) was added to each well according to the manufacturer’s instructions, respectively, which was changed every 2–3 days. 21 days later, the induction medium was removed, and the cells were washed with PBS at least 3 times. Then, after being fixed with 4% paraformaldehyde (PFA) (Servicebio, Wuhan, China) for 20 min at room temperature, the cells were stained with 1% alizarin red S (ARS) solution (Cyagen, Wuhan, China) to observe the deposition of mineralized nodules and stained with 2% oil red O solution (Cyagen, Wuhan, China) for determining lipid droplet formation under an inverted optical microscope (Olympus, Tokyo, Japan), and the representative images were captured. In addition, aimed at ascertaining the onset of DPSC odonto/osteogenic differentiation, total RNA was extracted from early odonto/osteogenic DPSCs at various time intervals using the SteadyPure Universal RNA Extraction Kit (Accurate, Changsha, China) following the manufacturer’s protocols. After analyzing RNA purity with a K5500 spectrophotometer (Kaiao, Beijing, China), cDNA was synthesized using an Evo M−MLV RT Kit (Accurate, Changsha, China). With the aim of assessing the gene expression level of *ALP* and *sex-determining region Y-box 2* (*SOX2*), real-time quantitative polymerase chain reaction (RT-qPCR) was conducted using the SYBR Green PCR Master Mix Kit (Accurate, Changsha, China) in an LC480II RT-qPCR system (Roche, Basel, Switzerland) with the following thermal cycling program: an initial heat activation step at 95 °C for 20 min followed by 40 cycles of denaturation at 95 °C for 3 s and annealing at 60 °C for 30 s. The sequences of primers are listed in Table S1.

In order to evaluate the chondrogenic capability of DPSCs, 3×10^5^ DPSCs were transferred to a centrifuge tube. Subsequently, chondrogenic induction medium (Cyagen, Wuhan, China) was added to the tube, which was then centrifuged at 150 g for 5 min. After discarding the supernatant, the induction medium was gently added to the tube for continuous cell cultivation. Once cell precipitation and aggregation occurred, the tube was carefully tapped to detach the cell clusters from the bottom and suspend them in the medium, which was replaced every 3 days. 21 days later, the cell clusters were retrieved and fixed in 4% PFA for 30 min. After paraffin embedding, the cell clumps were frozen and sectioned, and the formation of blue cartilage matrix was detected by toluidine blue staining.

For the detection of MSC-specific surface markers on DPSCs, 3×10^7^ DPSCs were resuspended in an appropriate amount of PBS, and 100 μL of single-cell suspension was added to separate flow tubes. Subsequently, fluorescent antibodies against negative marker CD34 and positive markers CD73, CD90, and CD105 (BD Science, San Jose, CA, USA) were added to each tube and incubated in the dark at room temperature for 30 min. After incubation, PBS was added to the flow tube and centrifuged at 1200 rpm for 5 min to wash off excess antibodies. The stained cells were then resuspended in PBS and quantified via a CytoFLEX flow cytometer (Beckman Coulter, Miami, FL, USA) followed by FlowJo software (Version 10.8.1, BD Science, San Jose, CA, USA) analysis.

### Extraction and identification of exosomes

DPSCs were inoculated into T175 cell culture flasks, then cultured with ordinary growth medium and osteogenic induction medium for 3 d, respectively. After the cultivation, the above two culture media were replaced with the exosome-free growth medium, and the supernatants of regular-cultured DPSCs and odontogenic-induced DPSCs were collected 48h later. Next, the supernatant was centrifuged at 2000 g for 30 min in a high-speed low-temperature centrifuge (Eppendorf, Hamburg, Germany) to remove cells and impurities, followed by centrifugation at 10,000 g for 30 min to remove cell debris. Subsequently, the newly collected supernatant was centrifuged at 100,000 g for 90 min in an Optima XE-90 ultracentrifuge (Beckman Coulter, Miami, FL, USA), and the supernatant was discarded to obtain DPSC-Exos and DPSC-Od-Exos. Then, the above two types of exosomes were resuspended in 100 μL of PBS and stored at −80 °C in an ultra-low temperature freezer for later use.

For exosome identification, transmission electron microscopy (TEM) (HITACHI, Tokyo, Japan) was utilized to observe the ultrastructure of DPSC-Exos and DPSC-Od-Exos. Then, based on the BCA Protein Assay Kit (Beyotime, Shanghai, China) instructions, the protein concentrations of DPSC-Exos and DPSC-Od-Exos were measured, and the representative protein markers of exosomes were detected by Western blotting (WB), including tumor susceptibility gene 101 (TSG101) and CD63 (ABclonal, Wuhan, China). Moreover, for the examination of exosome size and distribution, nanoparticle tracking analysis (NTA) was performed on the obtained exosomes using a ZetaView nanoparticle analyzer (Particle Metrix, Meerbusch, Germany) according to the manufacturer’s guidelines.

### Labeling and uptake of exosomes

According to the manufacturer’s instructions, the PKH26 (Umibio, Shanghai, China) working solution was prepared to a final concentration of 100 μM. Subsequently, DPSC-Exos and DPSC-Od-Exos were separately added to the PKH26 working solution and incubated for 10 min. Following the exosome isolation approach mentioned earlier, PKH26-stained exosomes were extracted again to remove excess dye and resuspended in 200 μL of PBS. Then, PKH26-labeled exosomes were added separately to DPSCs and HUVECs (iCell, Shanghai, China) and incubated for 24h to permit exosome internalization. After PBS washing, cells were fixed with 4% PFA for 20 min, stained for cytoskeleton using phalloidin (Abbkine, Wuhan, USA) and nuclei with 6-diamidino-2-phenylindole (DAPI) (Servicebio, Wuhan, China). Finally, after PBS washing, the cellular uptake of two types of exosomes was observed using a confocal laser scanning microscopy (CLSM) (Olympus, Tokyo, Japan), and images were captured.

### The effect of exosomes on cell proliferation and migration

For determining a suitable exosomal concentration for cell viability in the present investigation, DPSCs and HUVECs were initially seeded onto a 96-well plate at a density of 5×10^3^ cells/well and cultured overnight. Then, a series of concentration gradients of exosomes, including 5, 25, and 50 μg/mL, were arranged and cocultured with DPSCs and HUVECs. On days 1, 3, 5, and 7, 10% CCK-8 solution (MedChem Express, Monmouth Junction, NJ, USA) was added to each well following the manufacturer’s instructions and further incubated at 37 °C in the dark for 90 min. The changes in optical density (OD) at 450 nm were measured using a microplate reader (Molecular Devices, San Jose, CA, USA). As indicated by the results of the above experiments, cells were subsequently cultivated in the growth medium with or without exosomes according to the grouping design of the control group, 50 μg/mL DPSC-Exos and 50 μg/mL DPSC-Od-Exos. The CCK-8 assay was also conducted to directly compare the impact of DPSC-Exos and DPSC-Od-Exos on cell proliferative capability.

To explore the role of the above two types of exosomes in the migration of DPSCs and HUVECs, 1×10^4^ cells were plated into the upper chamber of a 24-well Transwell plate (Corning, New York, NY, USA). The growth medium with or without exosomes was added to the lower chamber according to the aforementioned grouping. After 24 and 48h coculture, the upper chamber was gently washed with PBS, and non-migrated DPSCs or HUVECs were removed from the upper side of the membrane with a sterile cotton swab. On the other hand, the migrated cells on the lower side of the membrane were fixed with 4% PFA and then stained with 0.1% crystal violet (Sigma-Aldrich, Saint Louis, CA, USA) for 10 min. Finally, the migrated cells were imaged using an inverted microscope (Olympus, Tokyo, Japan), and the cell number was counted for analysis.

### The effect of exosomes on DPSC odonto/osteogenic differentiation

According to the grouping above, DPSCs were cultured in osteogenic induction medium (Cyagen, Guangzhou, China) supplemented with or without exosomes. After 7 days, following the methods described earlier, the expression of *ALP*, *DMP-1, runt-related transcription factor-2* (*RUNX-2*), *bone morphogenetic protein-2* (*BMP-2*), *osteocalcin* (*OCN*), *collagen type I α* (*Col1α*), and *dentin sialophosphoprotein* (*DSPP*) was detected by RT-qPCR.

For ALP staining and activity assessment, DPSCs were subjected to odonto/osteogenic induction with or without exosomes for 7 days. Then, they were washed twice with PBS and fixed with 4% PFA at room temperature for 15 min. Afterward, the ALP staining solution was prepared following the instructions of a BCIP/NBT ALP Staining Kit (Beyotime, Shanghai, China) and added to DPSCs, which were incubated in the dark at room temperature for 1h. After washing away the excessive staining solution with PBS, the stained DPSCs were observed under an inverted microscope (Olympus, Tokyo, Japan). In addition, total proteins from odonto/osteogenically differentiated DPSCs were extracted using RIPA lysis buffer (Beyotime, Shanghai, China) on the same day of treatments. ALP protein content was determined by employing an ALP Quantitative Assay Kit (Jiancheng, Nanjing, China) according to the manufacturer’s protocols. The OD value at 520 nm was measured, and ALP activity was evaluated after normalizing to total proteins.

As mentioned in the previous part, DPSCs induced for odonto/osteogenic differentiation with or without exosomes for 21 days were stained using ARS solution (Cyagen, Wuhan, China), which were then examined under an inverted microscope (Olympus, Tokyo, Japan) for mineral nodule accumulation. Subsequently, to semi-quantitatively assess the calcium deposits, the calcified nodules were dissolved in 10% cetylpyridinium chloride (Yuanye, Shanghai, China) for 20 min, and the dissolved solution was collected for further OD value detection at 562 nm.

### The effect of exosomes on angiogenic potential of DPSCs and HUVECs

Following the experimental designs described before, DPSCs and HUVECs were cultured in angiogenic induction medium (iCell, Shanghai, China) with or without exosomes. After 7 days, total RNA was extracted, and RT-qPCR was administered to detect the expression of angiogenesis-related marker genes, including *angiopoietin II* (*ANG II*), *vascular endothelial growth factor* (*VEGF*), *platelet-derived growth factor A* (*PDGFA*), and *matrix metalloproteinase 9* (*MMP-9*).

For immunofluorescence staining, DPSCs or HUVECs were seeded in 6-well plates coated with coverslips. After 7 days of angiogenic induction, the coverslips were carefully removed, and cells grown on the coverslips were fixed with 4% PFA for 15 min. Next, the fixed cells were incubated with permeabilization solution (Beyotime, Shanghai, China) for 10 min and subjected to blocking buffer (Beyotime, Shanghai, China) for 30 min. Subsequently, the cells were cultured with primary antibodies against VEGF (1: 100) (Abclonal, Wuhan, China) overnight at 4 °C. After rinsing with PBS, the cells were incubated with goat anti-rabbit Cy3 secondary antibodies (1: 400) (ABclonal, Wuhan, China) at room temperature for 1h. Then, cells were separately stained with phalloidin for 30 min and DAPI for 5 min, and finally mounted with an anti-fluorescence quenching reagent (Solarbio, Beijing, China) to permit CLSM imaging for recording the immunofluorescence intensity of each group.

To perform in vitro tube formation angiogenesis assay, HUVECs and DPSCs that had been induced for angiogenic differentiation for 3 days as mentioned above were collected and cultivated at a density of 1×10^4^ cells/well onto a 96-well plate pre-coated with Matrigel (ABW Mogengel, Xiamen, China) at 37 °C. 6h later, the formation of vascular-like tube structures was captured by an inverted microscope (Olympus, Tokyo, Japan), and Image J software (Version 1.53t, National Institutes of Health, Bethesda, MD, USA) was employed to analyze and quantify the total tube length and the total length of branches as well as the number of nodes, junctions, branches, and meshes.

To evaluate the effect of two types of exosomes on the angiogenic potential of HUVECs and DPSCs in vivo, 500 μL of Matrigel loaded with cells and/or exosomes was divided into 4 distinct groups depending on the type of implanted cells: (1) Matrigel alone, (2) Matrigel supplemented with 1×10^7^ cells, (3) Matrigel integrated with cells and DPSC-Exos, and (4) Matrigel combined with cells and DPSC-Od-Exos. In consequence, a total of 8 experimental groups were established, comprising 4 groups dedicated to each cell type. Subsequently, under general anesthesia, the above systems were injected subcutaneously into both sides of the back of 6–8-week-old female BALB/C nude mice to form Matrigel plugs, and all mice were raised by certificated breeders at a specific pathogen-free laboratory animal research center. After 14 days, the mice were euthanized, and all Matrigel plugs were dissected, which were then fixed in 4% PFA, embedded in paraffin, and sectioned into slides. Based on the experimental methods described above, the sections were incubated with anti-VEGF (1: 100) (Abclonal, Wuhan, China) for immunofluorescence staining. Moreover, to implement immunohistochemical staining, CD31 primary antibodies (1: 100) (Abclonal, Wuhan, China) were added to sections and incubated overnight at 4 °C before being treated with horseradish peroxidase-conjugated secondary antibodies. Subsequently, for visualizing immune complexes, DAB chromogenic solution (Servicebio, Wuhan, China) was applied according to the manufacturer’s instructions. Lastly, cell nuclei were counterstained with hematoxylin dye. The staining results were determined by an optical microscope (Olympus, Tokyo, Japan) and analyzed for the number of VEGF- and CD31-positive tubular structures using Image J software (Version 1.53t, National Institutes of Health, Bethesda, MD, USA).

### The effect of exosomes on neurogenic differentiation of DPSCs

Experimental grouping was the same as above, and DPSCs were cultivated in neurogenic induction medium (Puhe, Wuxi, China) with or without exosomes. After 7 days, total RNA was extracted for RT-qPCR to examine the expression of neurogenesis-associated marker genes, including *Nestin* and *glial cell-derived neurotrophic factor* (*GDNF*).

Following previously mentioned cell immunofluorescence staining protocols, primary antibodies against Nestin (1: 500) (ABclonal, Wuhan, China) were added to neurogenically induced DPSCs, and cells were incubated at 4 °C overnight. After washing with PBS, cells were cultured with goat anti-rabbit Rhodamine secondary antibodies (1: 100) (ABclonal, Wuhan, China) at room temperature for 1h. Then, cells were treated with phalloidin and DAPI, respectively, and the immunofluorescence intensity of each group was recorded under CLSM.

### Subcutaneous transplantation of human tooth root fragments in mice

The roots of healthy premolars extracted for orthodontic purposes were sectioned at the cementoenamel junction. Root fragments with a height of 5 mm and an inner diameter of 2 mm were prepared after the superficial cementum was removed using sterile dental fissure burs. Then, the collected tooth root fragments were immersed in 17% ethylenediaminetetraacetic acid (EDTA) at room temperature for 10 min to remove the smear layer, followed by sequential treatment with iodophor and 5.25% sodium hypochlorite for 30 min and 15 min. After rinsing with sterile PBS, treated tooth root fragments were soaked in sterile PBS and incubated at 37 °C for 7 days to eliminate residual chemical disinfectants and potential microbial contamination.

Subsequently, Matrigel encapsulating DPSCs and/or exosomes was filled in the pulp cavity of each tooth root fragment. According to the composition of filling materials, the experiment was divided into 4 groups, including Matrigel, Matrigel+1×10^7^ DPSCs; Matrigel+DPSCs+DPSC-Exos, and Matrigel+DPSCs+DPSC-Od-Exos. The filled tooth root fragments were subcutaneously transplanted into the left and right dorsal regions of 6–8-week-old BALB/C female nude mice. After 8 weeks, the grafts were retrieved, fixed in 4% PFA, and decalcified in 10% EDTA. Then, paraffin-embedded tissue sections were subjected to hematoxylin-eosin (HE) staining with the assistance of the Experimental Medicine Research Center, Tongji Hospital, Tongji Medical College, Huazhong University of Science and Technology to observe the generation of new pulp-dentin complex under a light microscope.

To further validate the multifaceted roles of exosomes in pulp-dentin complex regeneration, immunofluorescence staining for odontogenic marker DSPP, angiogenic marker CD31, and neurogenic marker neurofilament 200 (NF200) was conducted separately. Based on the methods discussed before, tissue sections were incubated with primary antibodies anti-DSPP (1: 200) (ABclonal, Wuhan, China), anti-CD31 (1: 200), and anti-NF200 (1: 200) (ABclonal, Wuhan, China) at 4 °C. Then, secondary antibodies goat anti-rabbit 488 (1: 200) (ABclonal, Wuhan, China) and goat anti-rabbit Cy3 were added followed by DAPI staining. Finally, CLSM was manipulated to exhibit and capture the fluorescence intensity in each group. Fluorescence signals were quantified by measuring the percentage of the areas positive for DSPP, CD31, and NF200 using Image J software (Version 1.53t, National Institutes of Health, Bethesda, MD, USA).

### Statistical analysis

Statistical analysis was conducted with GraphPad Prism software (Version 9.0.0, San Diego, CA, USA). Data were presented as mean ± standard deviation from at least three independent experiments. Before applying parametric statistical tests, we performed normality testing on our data to assess its distribution using the Shapiro-Wilk test, supplemented by visual inspection through Q-Q plots and histograms. Student’s *t*-test was adopted for comparison between two groups and one-way ANOVA followed by Tukey’s test was employed to compare multiple groups. Different lower-case letters indicated statistically significant difference between groups at *p* < 0.05.

## Results

### DPSCs exhibited MSC characteristics and effectively released DPSC-Od-Exos and DPSC-Exos depending on culture conditions

**Fig. 1:**
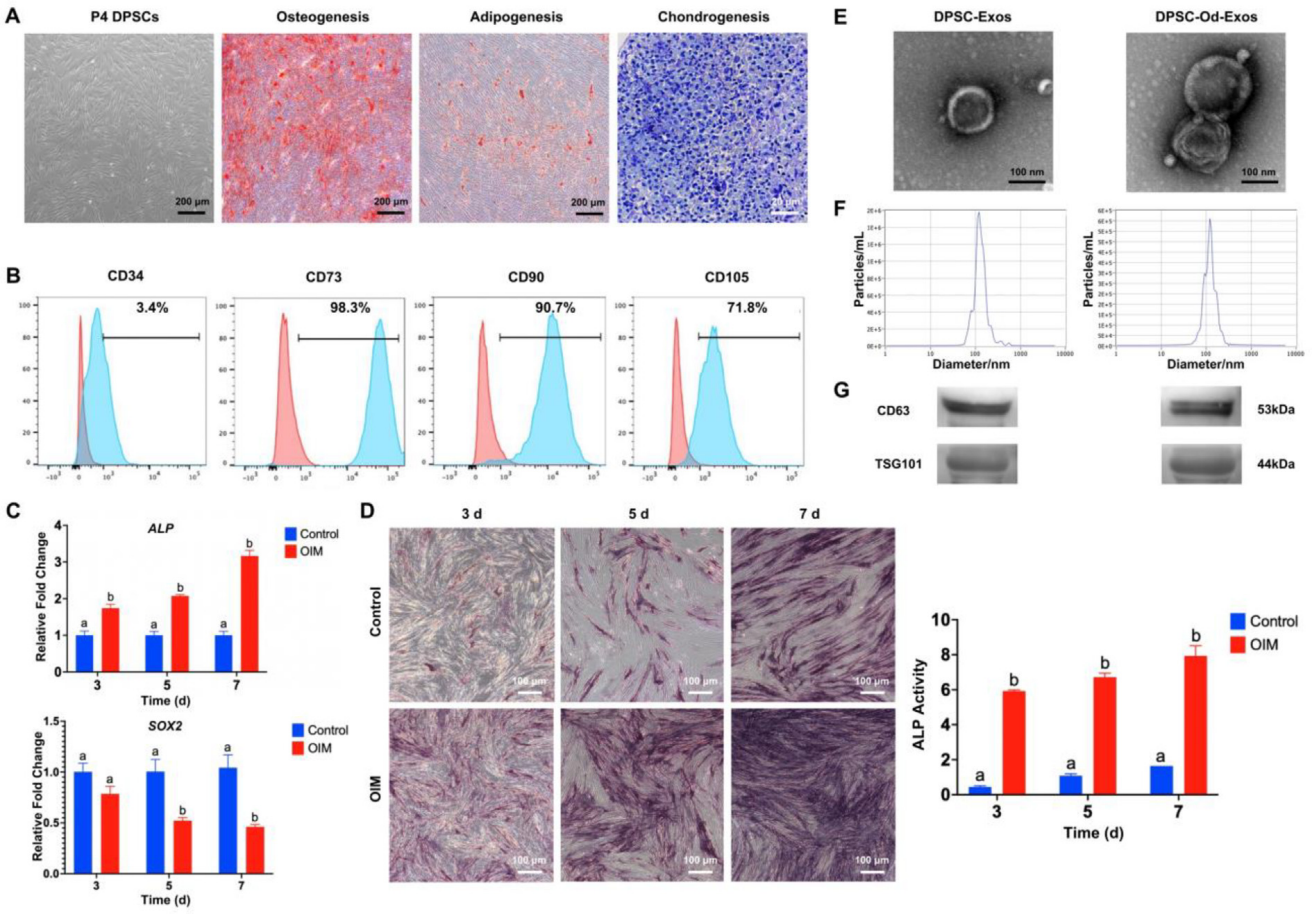
**Human DPSCs exhibited MSC attributes and secreted typical exosomes. (A, B) DPSCs were characterized as typical MSCs.** (**A**) P4 DPSCs adherent to the plastic surface displayed a spindle-like shape. In addition, DPSCs promoted the accumulation of orange-red calcified nodules, the formation of bead-like lipid droplets, and the production of light-blue extracellular matrix under proper induction. Scale bars: 200 μm and 20 μm (high magnification). (**B**) Flow cytometry signified that as opposed to the negatively expressed CD34, CD73, CD90, and CD105 were highly expressed in DPSCs. (**C, D**) When subjected to OIM, DPSCs initiated odonto/osteogenic differentiation in 3 days, although they still maintained MSC stemness. (**C**) Compared with *SOX2* gene expression without a marked difference at 3 days, the expression of *ALP* was substantially augmented. (**D**) Coincidently, ALP staining and activity evaluation confirmed that DPSCs initially triggered odonto/osteogenic differentiation in 3 days. (**E-G**) Regular DPSC-Exos and DPSC-Od-Exos obtained from DPSCs that underwent initial odontogenic differentiation for 3 days met the requirements of exosomes. (**E**) As detected by TEM, DPSC-Od-Exos and DPSC-Exos had similar morphological features and exhibited saucer-like microstructures. Scale bar: 100 nm. (**F**) It was denoted by NTA that DPSC-Od-Exos and DPSC-Exos, ranging from 107.2 nm to 151.1 nm, had an average diameter of around 130 nm. (**G**) The WB assessment showed that DPSC-Od-Exos and DPSC-Exos were highly enriched in transmembrane protein CD63 and cytoplasmic protein TSG101, and no difference was notably distinguished, conforming the characteristics of exosomes. Noted: Different letters above the bars indicated statistically significant difference at *p* < 0.05.

Dedicated to elucidating the potential of DPSC-Od-Exos in reshaping a suitable regenerative microenvironment for pulp-dentin complex engineering, we first isolated DPSCs through enzymatic hydrolysis of tissue blocks. As visualized under a light microscope, P4 DPSCs were adherent to the plastic surface and showed a typical spindle shape (Fig. 1A). When exposed to osteogenic differentiation medium, DPSCs gave rise to the deposition of orange-red mineralized nodules, which were distributed extensively in sheets and plaques (Fig. 1A). After adipogenic induction, bead-like lipid droplets were identified in DPSCs, indicative of the presence of adipocytes (Fig. 1A). Besides, chondrogenic induction contributed to the formation of light-blue cartilage-like extracellular matrices, suggesting that DPSCs were capable of differentiating into chondrocytes (Fig. 1A). Collectively, DPSCs held immense potential for multilineage differentiation. In addition, regarding the expression of cell surface markers, flow cytometry indicated that in sharp contrast with CD34, which was almost negatively expressed on DPSCs, the expression rates of positive markers CD73, CD90, and CD105 were 98.3%, 90.7%, and 71.8%, respectively (Fig. 1B). Hence, based on the plastic adherence, trilineage differentiation capacity, and specific marker expression, we verified that DPSCs obtained in the current study possessed the characteristics of MSCs, laying a solid foundation for subsequent exosome extraction and cell function research.

Next, in an attempt to collect DPSC-Od-Exos, DPSCs were subjected to osteogenic induction, and it was confirmed that 5 days later, the mRNA expression of *SOX2*, which determined the multipotency of stem cells, was dramatically inhibited, while 3 days later, the expression level of *ALP* was considerably elevated (Fig. 1C). In accordance with these, ALP staining further demonstrated that on the 3rd day of osteogenic induction, the intracellular accumulation of black-blue precipitates was substantially enhanced (Fig. 1D, left panel), which was also ascertained by markedly increased ALP activity (Fig. 1D, right panel), emphasizing that DPSCs were able to initiate odonto/osteogenic differentiation in 3 days although they still maintain MSC stemness. Accordingly, we harvested DPSC-Od-Exos from the supernatant of DPSCs that underwent early odontogenic differentiation for 3 days. According to TEM examination, DPSC-Od-Exos and DPSC-Exos displayed saucer-like microstructures with lipid bilayer membranes, and no remarkable difference in shape was observed between them (Fig. 1E). Moreover, DPSC-Od-Exos shared a similar size distribution with DPSC-Exos, ranging from 107.2 nm to 151.1 nm, with an average diameter of around 130 nm (Fig. 1F). Of importance, as validated by WB, both exosomes were highly enriched in transmembrane protein CD63 and cytoplasmic protein TSG101, and no difference was notably distinguished (Fig. 1G). To sum up, DPSC-Od-Exos and DPSC-Exos acquired in the present study conformed to the essential characteristics of exosomes, paving the way for exploring their roles in orchestrating the fate of DPSCs and HUVECs.

### DPSC-Od-Exos drastically promoted odontogenic differentiation of DPSCs in vitro

**Fig. 2:**
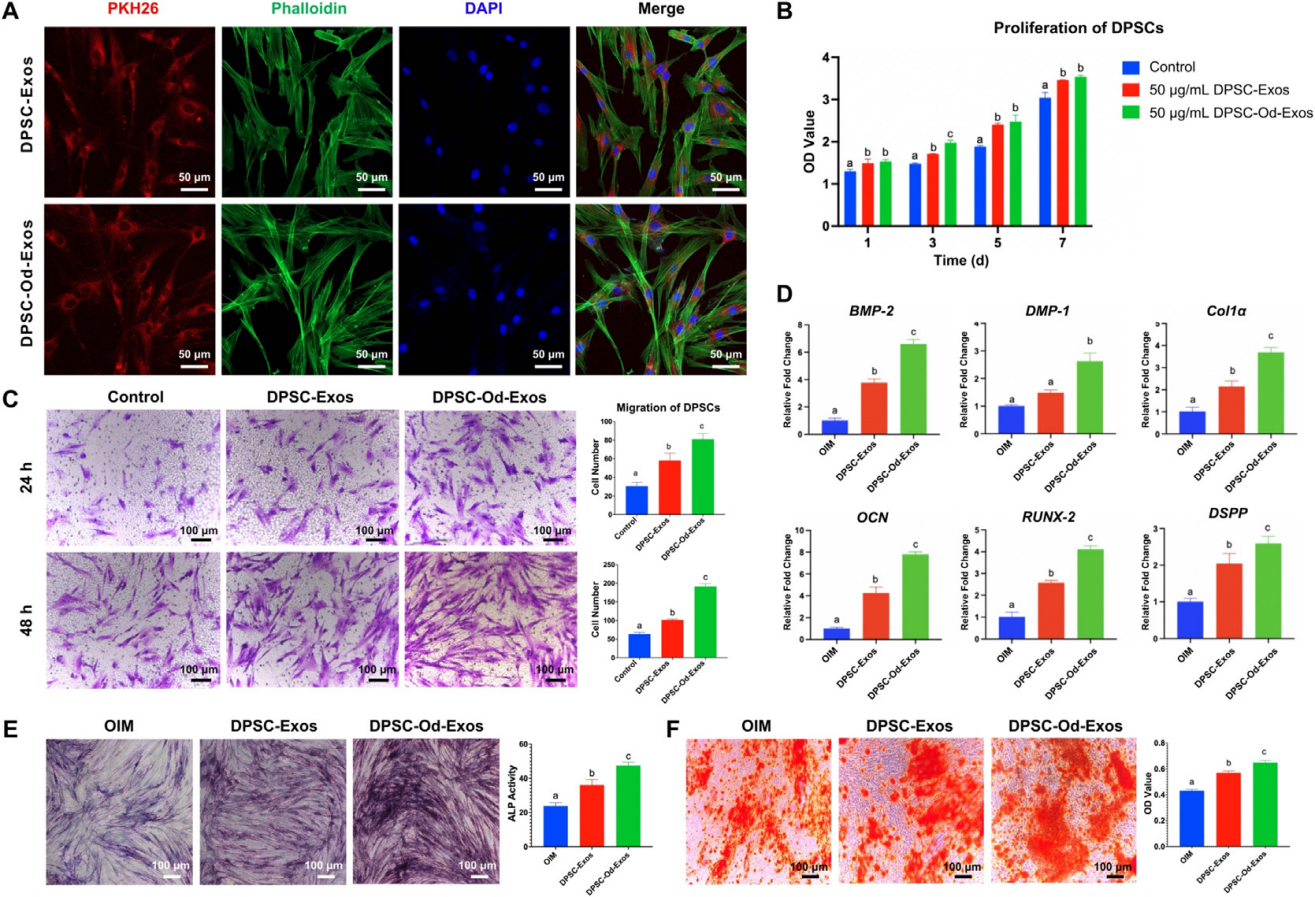
**DPSC-Od-Exos were dramatically beneficial for the odontogenic differentiation of DPSCs in vitro.** (**A**) As confirmed by the uptake assay, DPSC-Od-Exos and DPSC-Exos positively stained by PKH26 could be effectively internalized by DPSCs, thus leading to extensive distribution in the cytoplasm. Scale bar: 50 μm. (**B**) The application of DPSC-Od-Exos and DPSC-Exos had similar efficacy and triggered a significantly enhanced proliferation of DPSCs. (**C**) Compared with DPSC-Exos, DPSC-Od-Exos could time-dependently promote the migratory capacity of DPSCs, as suggested by the remarkably increased number of migrated cells. Scale bar: 100 μm. (**D**) It was elucidated by RT-qPCR that, in contrast with DPSC-Exos, DPSC-Od-Exos could elicit an outstanding enhancement in the expression of odonto/osteogenic-correlated genes, such as *DSPP*, *DMP*-1, and *OCN*. (**E**) In line with gene expression, DPSC-Od-Exos were more potent than DPSC-Exos in facilitating ALP staining and activity. Scale bar: 100 μm. (**F**) By performing ARS staining, we further illustrated that DPSC-Od-Exos notably fostered the deposition of orange-red mineralized nodules, therefore contributing to a higher OD value in semi-quantification analysis. Scale bar: 100 μm. Noted: Different letters above the bars indicated statistically significant difference at *p* < 0.05.

Aiming to unveil the potential of DPSC-Od-Exos in ameliorating DPSC functions, we first performed an uptake assay. Immunofluorescence staining revealed that PKH26-labeled DPSC-Od-Exos and DPSC-Exos could be enormously internalized by DPSCs, contributing to their intense and widespread distribution between the plasma membranes and nuclei (Fig. 2A). As depicted by the CCK-8 assay, both DPSC-Od-Exos and DPSC-Exos could dose-dependently ameliorate the viability of DPSCs (Fig. S1A). In this regard, 50 μg/mL was considered as an appropriate concentration and employed for the subsequent experiments. Encouragingly, DPSC-Od-Exos and DPSC-Exos at 50 μg/mL elicited a robust promoting effect on DPSC proliferation, and marked difference was not identified between them with time elapsing (Fig. 2B). In terms of migratory competence, compared with DPSC-Exos, the introduction of DPSC-Od-Exos could significantly augment the number of migrated DPSCs in a time-dependent manner (Fig. 2C). Then, special attention was paid to the impact of DPSC-Od-Exos on DPSC odontogenic differentiation. Despite the fact that DPSC-Od-Exos and DPSC-Exos were capable of facilitating odonto/osteogenic-related gene expression, DPSC-Od-Exos possessed a much stronger potential than DPSC-Exos (Fig. 2D). In line with this, it was elaborated that in comparison with DPSC-Exos, DPSC-Od-Exos potentiated the ALP staining and activity (Fig. 2E). Additionally, according to ARS staining, the employment of DPSC-Od-Exos was more beneficial than DPSC-Exos for the accumulation of orange-red mineral nodules, which was also confirmed by semi-quantification evaluation (Fig. 2F). Taken together, the above findings suggested that emerging as biomimetic nanotherapeutic tools, DPSC-Od-Exos have broad prospects to orchestrate DPSC fate and promote dentin regeneration in vivo.

### DPSC-Od-Exos considerably facilitated endothelial and neurogenic differentiation of DPSCs in vitro

**Fig. 3:**
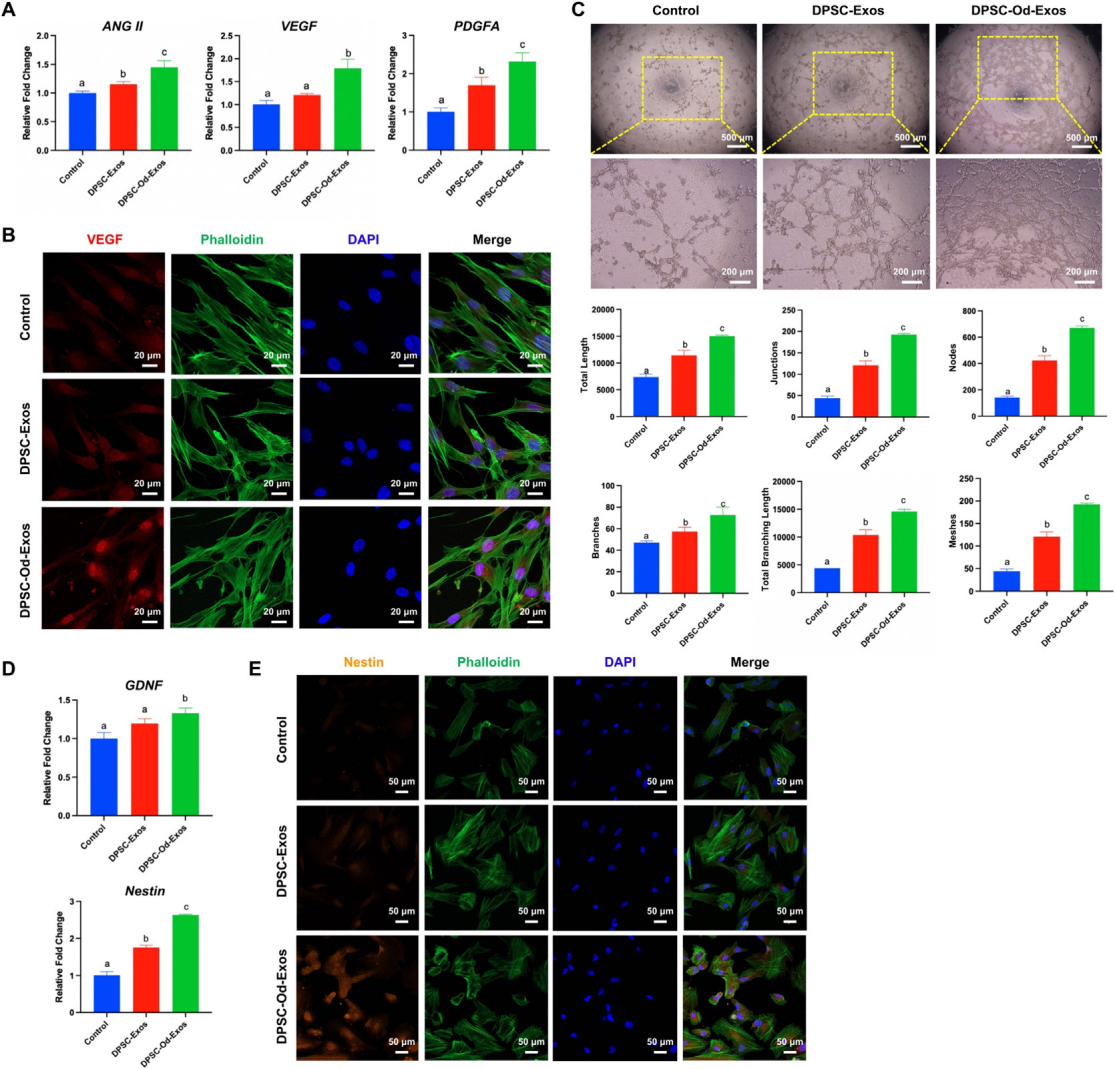
**DPSC-Od-Exos were capable of inducing endothelial and neurogenic differentiation of DPSCs in vitro.** (**A-C**) DPSC-Od-Exos exerted a notable promoting effect on DPSC endothelial cell differentiation. (**A**) In contrast with DPSC-Exos, DPSC-Od-Exos enormously enhanced the expression level of angiogenesis-related genes, including *ANG II*, *VEGF*, and *PDGFA*. (**B**) Consistently, DPSC-Od-Exos were more suitable inducers in upregulating the immunostaining intensity of VEGF compared with DPSC-Exos. Scale bar: 20 μm. (**C**) The tube formation assay indicated that as opposed to blank control that presented disorganized networks, both DPSC-Od-Exos and DPSC-Exos were dedicated to forming regular tubular-like structures. However, in sharp contrast with DPSC-Exos, DPSC-Od-Exos contributed to producing capillary-like networks in an orderly manner, thus drastically enhancing the total length, total branching length, and the number of junctions, nodes, branches, and meshes. Scale bars: 500 μm and 200 μm (high magnification). (**D, E**) DPSC-Od-Exos possessed substantial benefits in stimulating the neurogenic differentiation of DPSCs. (**D**) As depicted by RT-qPCR, the expression of neurogenic-associated markers GDNF and Nestin was massively strengthened by DPSC-Od-Exos. (**E**) In accordance with this, DPSC-Od-Exos had obvious superiority over DPSC-Exos in enhancing the immunostaining of Nestin. Scale bar: 50 μm. Noted: Different letters indicated statistically significant difference (*p* < 0.05).

Focused on the feasibility of DPSC-Od-Exos to induce DPSC endothelial and neurogenic differentiation, we conducted RT-qPCR to determine those associated mRNA expressions. Intriguingly, the levels of angiogenic markers, including *ANG II*, *VEGF*, and *PDGFA*, were substantially boosted after the application of DPSC-Od-Exos (Fig. 3A). It was also noteworthy that in contrast with normal DPSC-Exos, DPSC-Od-Exos exerted a more reliable and powerful influence on the immunostaining of VEGF, thus reinforcing VEGF expression intensity (Fig. 3B). Consistent with these results, we discovered that DPSC-Od-Exos displayed a superior efficacy on DPSCs for tubular-like structure formation, as verified by remarkably elevated numbers of junctions, nodes, branches, and meshes as well as total length and total branching length (Fig. 3C). Consequently, DPSC-Od-Exos showed desirable performance in initiating the endothelial cell differentiation of DPSCs, which amplified their therapeutic value in pulp angiogenesis.

Subsequently, a massive effort was made to assess the role of DPSC-Od-Exos in neurogenesis. From the perspective of gene level, there was an abundant expression of *GDNF* and *Nestin* for DPSCs pretreated with DPSC-Od-Exos (Fig. 3D). Notably, from the protein perspective, we illuminated via immunostaining of Nestin that DPSC-Od-Exos exhibited a better stimulating impact on the expression of Nestin compared with DPSC-Exos (Fig. 3E), shedding light on the promising benefits of DPSC-Od-Exos in inducing the recovery of pulp neural function.

### DPSC-Od-Exos remarkably enhanced angiogenic potential of HUVECs in vitro

**Fig. 4:**
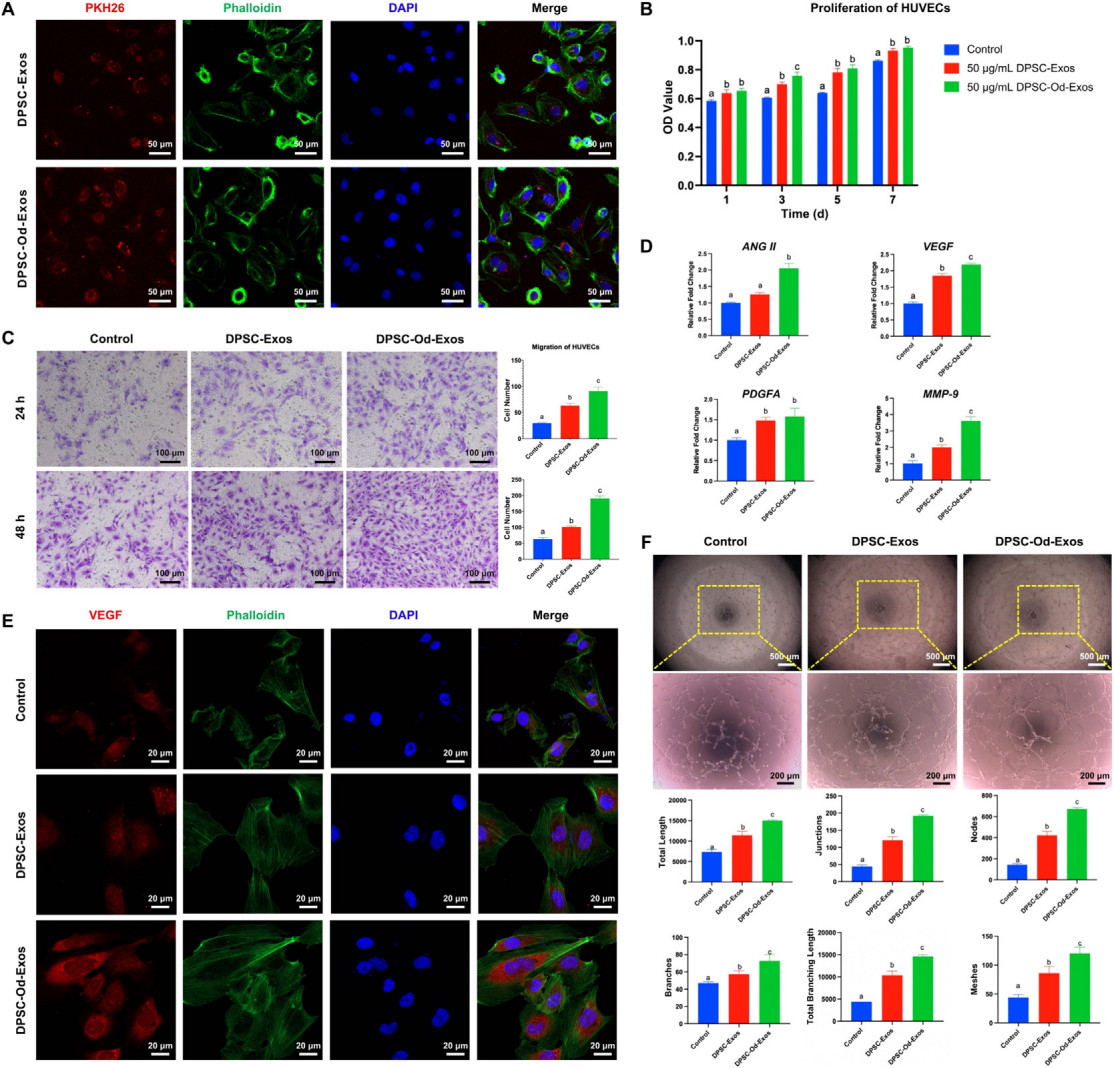
**DPSC-Od-Exos exhibited more robust promoting potential in the angiogenesis of HUVECs in vitro.** (**A**) The uptake assay verified that DPSC-Od-Exos and DPSC-Exos could be extensively taken up by HUVECs, resulting in a wide distribution of red fluorescence in the HUVEC cytoplasm. Scale bar: 50 μm. (**B**) As demonstrated by the CCK-8 assay, DPSC-Od-Exos and DPSC-Exos at 50 μg/mL time-dependently potentiated the proliferative property of HUVECs. **(C**) Concentrated on the migratory capability of HUVECs, we discovered that DPSC-Od-Exos could be more effective than DPSC-Exos in elevating the number of migrated cells in a time-dependent manner. Scale bar: 100 μm. (**D**) According to RT-qPCR, compared with DPSC-Exos, DPSC-Od-Exos considerably promoted the expression level of angiogenic genes, including *ANG II*, *VEGF*, *PDGFA*, and *MMP-9*. (**E**) The immunostaining against VEGF delineated that the expression intensity was substantially facilitated after the employment of DPSC-Od-Exos. Scale bar: 20 μm. (**F**) It was clarified through tube formation assay that although DPSC-Od-Exos and DPSC-Exos were able to enhance the production of regularly organized tubular structures, DPSC-Od-Exos exhibited better performance than DPSC-Exos, remarkably increasing the number of junctions, nodes, branches, and meshes as well as the total length and total branching length. Scale bars: 500 μm and 200 μm (high magnification). Noted: Different letters indicated statistically significant difference between groups (*p* < 0.05).

Before disclosing whether DPSC-Od-Exos played a positive role in the angiogenic process of HUVECs, we needed to figure out their uptake efficiency by HUVECs. It was evidenced that after incubation with HUVECs, PKH26-labeled DPSC-Od-Exos and DPSC-Exos were localized into the cytoplasm of HUVECs (Fig. 4A), providing a deeper insight into exosome-mediated cellular behavior regulation. In this regard, by performing the CCK-8 assay, we confirmed exosomes at 50 μg/mL as the desirable concentration in the following investigations (Fig. S1B). Moreover, it was observed that both DPSC-Od-Exos and DPSC-Exos at 50 μg/mL could time-dependently stimulate the proliferation of HUVECs and present a similar efficacy (Fig. 4B). As shown by the Transwell assay, DPSC-Od-Exos preferentially possessed a strong promigratory influence on HUVECs, extraordinarily increasing the number of HUVECs transmigrating through the membrane (Fig. 4C). In particular, a time-dependent fashion could also be established. With respect to angiogenic marker expression, DPSC-Od-Exos elicited a more desirable effect on the promotion of *ANG II*, *VEGF*, *PDGFA*, and *MMP-9* in comparison with DPSC-Exos, underlining the robust proangiogenic potential of DPSC-Od-Exos (Fig. 4D). Furthermore, it was worth mentioning that under a confocal microscope, the immunofluorescence expression of VEGF was dramatically facilitated as a consequence of DPSC-Od-Exo administration (Fig. 4E). Concomitantly, as validated by the tube formation assay, DPSC-Od-Exos were capable of optimizing the construction of capillary-like networks, substantially increasing junctions, nodes, branches, etc (Fig. 4E). Together, compared with DPSC-Exos, DPSC-Od-Exos could be acknowledged as more prospective mediators of ameliorating HUVEC biological behaviors and advancing their angiogenic process.

### DPSC-Od-Exos contributed to abundant vascular reconstitution after immersing in Matrigel plugs

**Fig. 5:**
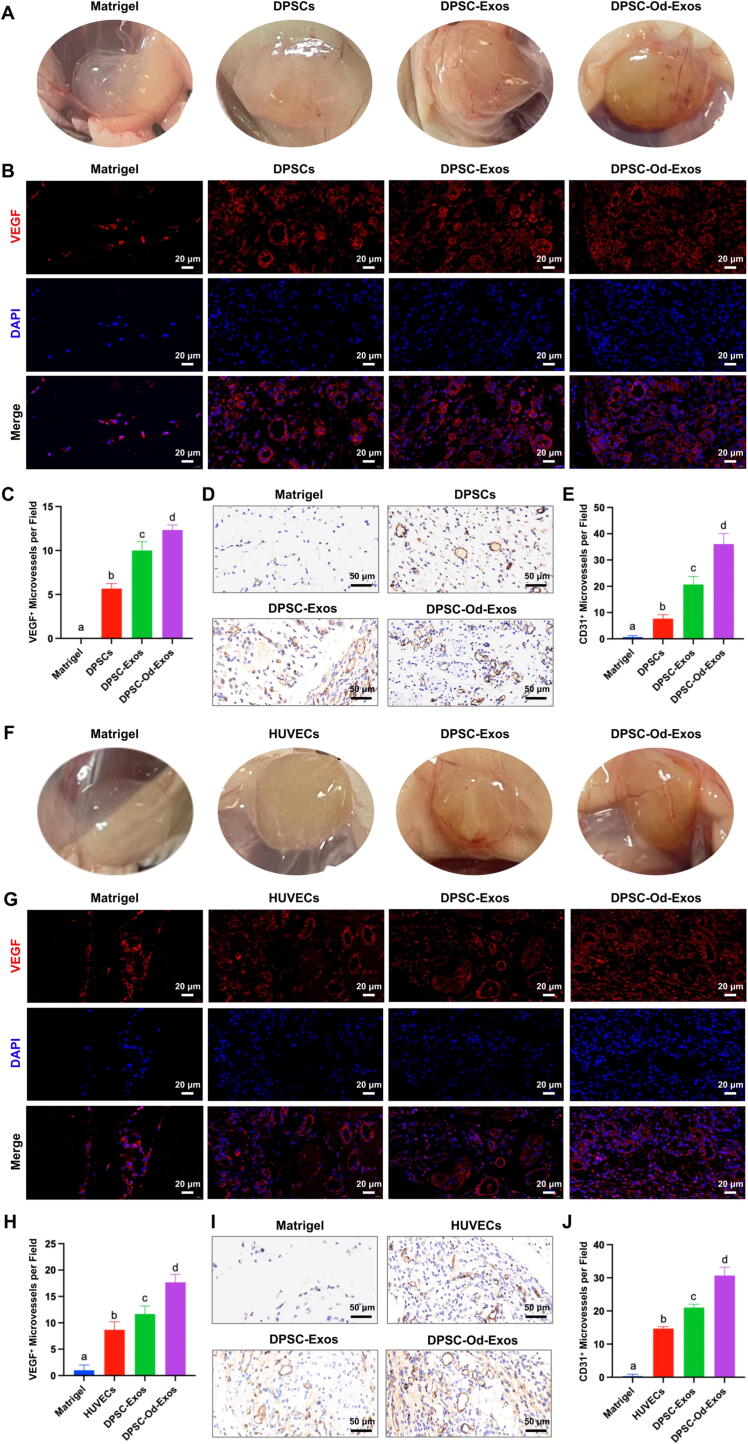
**DPSC-Od-Exos performed better than DPSC-Exos in enhancing angiogenesis when incubated with DPSCs or HUVEVs in Matrigel plugs.** (**A-E**) The combined application of DPSC-Od-Exos and DPSCs remarkably contributed to the formation of abundant capillary-like structures. (**A**) In visual observation, a more apparent reddish-brown appearance could be identified following the introduction of DPSC-Od-Exos. (**B, C**) As revealed by immunofluorescence staining against VEGF, DPSC-Od-Exos were more effective than DPSC-Exos in promoting the formation of positively stained tubular networks. Scale bar: 20 μm. (**D, E**) In accordance with the above findings, DPSC-Od-Exos elicited a better angiogenic performance in facilitating the expression of CD31, suggesting that DPSC-Od-Exos were capable of inducing DPSC endothelial cell differentiation. Scale bar: 50 μm. (**F-J**) Compared with DPSC-Exos, DPSC-Od-Exos considerably enhanced the angiogenic potential of HUVECs. (**F**) By visual inspection, DPSC-Od-Exos showed notable advantages in stimulating microvessel formation for HUVECs, thus giving rise to red coloration. (**G, H**) Under a confocal microscope, DPSC-Od-Exos were superior to DPSC-Exos in increasing the number of VEGF-positive vascular lumens. Scale bar: 20 μm. (**I, J**) It was evidenced through immunohistochemical assessment that the immunoactivity of CD31 to newly formed capillary tube-like networks was markedly promoted by DPSC-Od-Exos when compared with DPSC-Exos (**I**), which was also confirmed by quantification analysis (**J**). Scale bar: 50 μm. Noted: Different letters indicated statistically significant difference (*p* < 0.05).

In an attempt to confirm the dual functions of DPSC-Od-Exos in promoting endothelial cell differentiation of DPSCs and enhancing the angiogenic property of HUVECs, DPSCs or HUVECs immersed in Matrigel were subcutaneously implanted with DPSC-Od-Exos into the subcutaneous space of nude mice. As opposed to blank Matrigel and sole transplantation of DPSCs, which showed a pale white, an apparent reddish-brown color change could be observed by visual inspection for the combinatorial transplantation of DPSCs and DPSC-Od-Exos or DPSC-Exos (Fig. 5A), indicating that more abundant and intense vascular networks were produced. However, it was crucial to point out that DPSC-Od-Exos were superior to DPSC-Exos in strengthening the red coloration and enriching the number of blood vessels. By implementing immunofluorescence staining for VEGF, we further discovered that the synergistic application of DPSC-Od-Exos and DPSCs markedly enhanced the formation of vascular structures positively stained by VEFG red fluorescence (Fig. 5B-C). In accordance with these findings, as revealed by immunohistochemical staining of CD31, the number of CD31^+^ capillary-like microvessels was also considerably increased after the introduction of DPSC-Od-Exos (Fig. 5D-E), ensuring that DPSC-Od-Exos exhibited enormous potential to induce endothelial differentiation of DPSCs.

On the other hand, in response to DPSC-Od-Exo pretreatment, the extracted Matrigel plugs incorporating HUVECs showed a more noticeable reddish appearance in visual observation (Fig. 5F). In addition, it was evidenced through confocal microscopy detection that large amounts of VEGF^+^ tubular structures were distributed broadly after employing DPSC-Od-Exos, which displayed a remarkable difference with DPSC-Exos (Fig. 5G-H). Consistently, although DPSC-Od-Exos and DPSC-Exos elicited a positive regulation of CD31 expression, DPSC-Od-Exos were more conducive than DPSC-Exos to facilitate angiogenic property of HUVECs and augment the number of CD31^+^ tubular lumens (Fig. 5I-J). Hence, these findings indicated that in contrast with DPSC-Exos, DPSC-Od-Exos could be recognized as novel and favorable proangiogenic factors to restore pulp angiogenesis.

### The combinatorial transplantation of DPSC-Od-Exos and DPSCs promoted total pulp-dentin complex regeneration in vivo

**Fig. 6:**
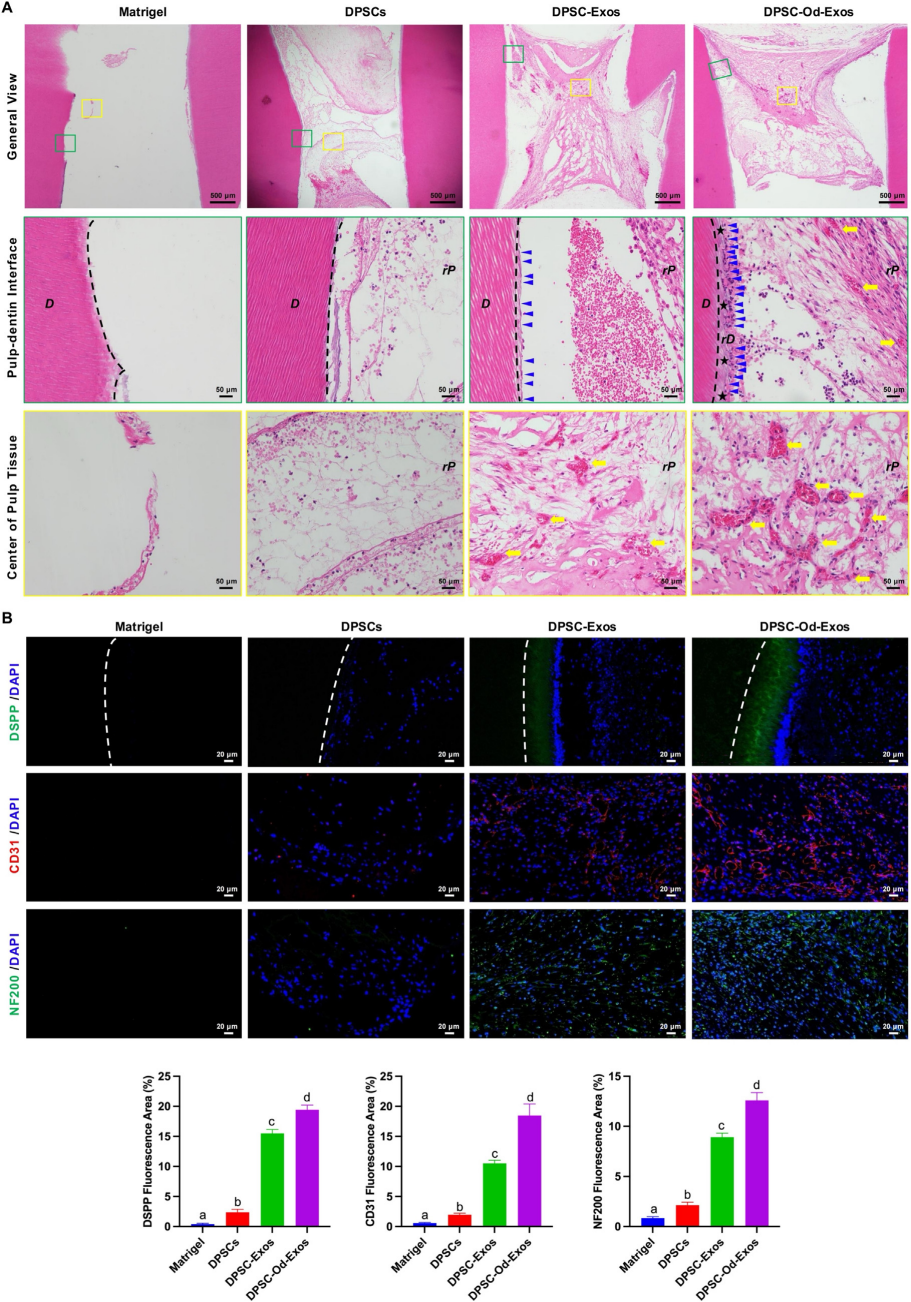
**DPSC-Od-Exos promoted complete regeneration of pulp-dentin complex by enhancing DPSC odontogenesis, angiogenesis, and neurogenesis.** (**A**) As demonstrated by HE staining, the sole DPSC transplantation led to the formation of disorganized soft tissue in the root canal. Notably, following the introduction of DPSC-Od-Exos and DPSC-Exos, pulp-like tissue with higher cell density and more regular structure could be observed. In contrast with the DPSC-Exo-treated group, DPSC-Od-Exos contributed to the formation of vascular-enriched pulp tissue and continuous tubular dentin layer with the alignment of odontoblast-like cells. Scale bar: 50 μm. The dashed line: the interface between original dentin and regenerated dentin; *D*: original dentin; *rD*: regenerated dentin; *rP*: regenerated pulp; black star: tubular dentin; blue arrowhead: odontoblast-like cells; yellow arrow: blood vessels. (**B**) According to immunofluorescence staining and quantification analysis, compared with DPSC-Exos, DPSC-Od-Exos displayed more robust potential to promote the deposition of DSPP-positive tubular dentin, the reconstitution of CD31-positive vascular networks, and the sprouting of NF200-positive sensory nerve fibers, indicating their promising versatile roles in inducing functional pulp-dentin complex regeneration. Scale bar: 20 μm. The dashed line: the interface between original dentin and regenerated dentin. Noted: Different letters above the bars indicated statistically significant difference at *p* < 0.05.

Whether DPSC-Od-Exos enabled complete regeneration of pulp-dentin complex remained to be elucidated. To this end, DPSC-Od-Exos and DPSCs encapsulated in Matrigel were injected into pretreated tooth root fragments and then subcutaneously transplanted into nude mice for 8 weeks. As revealed by HE staining, compared with blank Matrigel, the transplantation of DPSCs contributed to the formation of loose connective tissue in the pulp cavity, which, however, was deficient in the structural features of pulp-dentin complex (Fig. 6A). In particular, the combination of DPSC-Exos and DPSCs promoted the generation of vascularized pulp-like tissue with a limited number of odontoblast-like cells attached to the existing dentin wall (Fig. 6A). More importantly, in striking contrast with DPSC-Exos, DPSC-Od-Exos possessed a tremendously enhanced potential to induce the accumulation of tubular dentin on the surface of natural dentin, accompanied by a continuous layer of odontoblast-like cells extending their cytoplasmic projections into the newly formed dentinal tubules (Fig. 6A, rightmost panel). Additionally, abundant and dense vascular networks with relatively expanded lumens had been established in the pulp-like tissue.

**Fig. 7:**
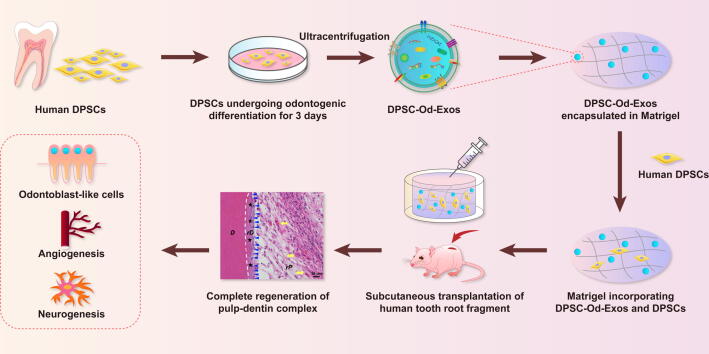
**Schematic illustration of the multifaceted roles of DPSC-Od-Exos in pulp-dentin complex regeneration.** DPSC-Od-Exos were obtained from DPSCs that initially triggered odontogenic differentiation via ultracentrifugation. After encapsulation in Matrigel with DPSCs, DPSC-Od-Exos were injected into the pulp cavity of human tooth root fragments and then subcutaneously implanted into nude mice. As revealed by histological evaluation, DPSC-Od-Exos contributed to the regeneration of complete pulp-dentin complex, which encompassed enriched neurovascular structures and a continuous layer of odontoblast-like cells extending their cytoplasmic projections into the newly formed dentinal tubules.

In view of this, we subsequently conducted immunofluorescence staining to determine the expression of DSPP, CD31, and NF200, which were intimately concerned with odontogenesis, angiogenesis, and neurogenesis, respectively. As depicted by DSPP staining, the newly generated dentin layer labeled with green fluorescence could be observed after introducing DPSC-Od-Exo and DPSC-Exos (Fig. 6B). Nevertheless, the intensity and scope of DSPP staining were considerably enhanced for DPSC-Od-Exos when compared with DPSC-Exos. Moreover, in the DPSC-Od-Exo-treated group, a continuous layer of DAPI-stained cells resembling odontoblasts was identified aligning to the freshly deposited dentinal wall, underscoring that DPSC-Od-Exos were more appropriate inducers of DPSC odontogenic differentiation. It was worthwhile to note that the formation of capillary-like tubular structures stained by CD31 was substantially facilitated with the involvement of DPSC-Od-Exos, thereby resulting in a remarkable difference with those conditioned with DPSC-Exos (Fig. 6B). In addition, it was confirmed that following NF200 staining, DPSC-Od-Exos exhibited better performance than DPSC-Exos in promoting the outgrowth of sensory nerve fibers (Fig. 6B), indicating that DPSC-Od-Exos were capable of stimulating DPSC neurogenesis and nerve spouting, which was beneficial for the recovery of pulp sensory function. These results were also illustrated by quantitative analysis of positively stained immunofluorescence areas (Fig. 6B). Consequently, considering the appearance of DSPP-positive neodentin, CD31-positive vascular networks, and NF200-positive nerve fibers, multifunctional DPSC-Od-Exos could be proposed as an innovative therapeutic strategy to optimize the local microenvironment for complete regeneration of functional pulp-dentin complex (Fig. 7).

## Discussion

Dental pulp tissue engineering represents a multidisciplinary field that integrates stem cell medicine, biomaterials, and molecular biology. Strictly speaking, it extends beyond the mere structural regeneration of a single tissue type. It comprises the regeneration of both pulp (soft tissue) and tubular dentin (hard tissue) in order to restore the functional integrity of pulp-dentin complex, which is imperative for nutritional support, sensory transduction, and immune response. Therefore, in the context of clinical endodontic dentistry, achieving complete functional regeneration of pulp-dentin complex has long been a desirable yet challenging goal. With the aim of creating a suitable microenvironment for dental pulp regeneration, traditional tissue engineering strategies predominantly rely on the administration of various growth factors. Nonetheless, the clinical translation of these approaches raises a myriad of concerns, particularly regarding the complexity of formulations. Consequently, the clinical efficacy of conventional growth factor-based pulp regeneration strategies has been markedly restricted. In contrast, exosomes derived from MSCs are gaining recognition as biomimetic nanoscale particles that can circumvent the shortcomings of traditional approaches and have gradually developed into a new promising avenue to reconstruct the regenerative microenvironment. In addition, the clinical application of exosomes presents multiple advantages, as emphasized previously. Hence, this study was dedicated to establishing a clinically predictable, highly feasible, and easily transformable pulp regeneration strategy utilizing exosomes to achieve both structural regeneration and functional reconstruction of pulp-dentin complex.

By mimicking and recapitulating developmental processes, developmental tissue engineering has emerged as a significant paradigm shift from traditional tissue engineering. In the field of pulp-dentin complex regeneration, researchers have increasingly advocated for the implementation of developmental engineering approaches. As revealed by Guo et al. in 2021, the bioengineered teeth, constructed from natural decellularized tooth matrix and deciduous DPSC aggregates, could effectively simulate the odontogenesis-associated developmental microenvironment upon implantation into the alveolar bones of minipig and promote the regeneration of functional dental pulp and periodontal tissue, characterized by the presence of neurovascular structures, primarily through the transportation of exosomes [8]. Furthermore, the pilot clinical trial conducted by the same research group clarified that the autologous implantation of bioengineered teeth contributed to the continued development and maturation of tooth roots, devoid of any detrimental side effects. These results indicated that developmental microenvironment engineering as an advanced transformative strategy elicited beneficial effects on tooth organ regeneration. Based on the principles of developmental engineering, the present study aimed to obtain odontogenic exosomes with multipotential capabilities for odontogenesis, angiogenesis, and neurogenesis by inducing odontogenic differentiation in DPSCs, thereby establishing a conducive microenvironment for pulp regeneration. To this end, by comparing DPSCs undergoing various durations of odontogenic differentiation through ALP activity and RT-qPCR, we identified that DPSCs subjected to odonto/osteogenic induction for 3 days have initially triggered odontogenic differentiation while retaining a degree of multipotency. In light of these observations, we proposed that exosomes derived from DPSCs that underwent odonto/osteogenic differentiation for 3 days might hold great promise as biomimetic therapeutic agents in regenerative endodontics. Subsequently, we extracted regular DPSC-Exos and odontogenic DPSC-Od-Exos via differential centrifugation technique. Consistent with previously published literature, the isolated DPSC-Exos and DPSC-Od-Exos exhibited a characteristic saucer-like morphology, shared a similar size distribution with an average diameter of approximately 130 nm, and expressed specific proteins CD63 and TSG101. Importantly, no notable difference was distinguished between the two exosome populations. These findings paved the way for further investigation into the impact of DPSC-Exos and DPSC-Od-Exos on pulp regeneration.

In clinical scenarios, the mechanical and chemical removal of infected dentin during root canal preparation inevitably diminishes dentin thickness of the root canal wall, thereby increasing the risk of tooth fracture, especially in young permanent teeth that have not yet fully developed. The regeneration of tubular dentin plays crucial roles in protecting internal susceptible pulp tissue, conducting external stimulating signals, and strengthening tooth stability. According to Shi et al. (2023), exosomes derived from immortalized embryonic stem cells exhibited a multifaceted capacity to enhance dental pulp cell functions, including migration, proliferation, and odontogenic differentiation through CD73/NT5E-mediated adenosine activation of AKT/ERK signaling pathways [24]. Intriguingly, these exosomes robustly promoted the regeneration of pulp-dentin-like tissue in both rat pulp exposure models and subcutaneously transplanted human premolar models. Also of note, it was confirmed in 2023 by Wang et al. that after subcutaneous implantation in thermosensitive chitin-based hydrogels containing DPSCs, DPSC-Exos potentiated the regeneration of pulp-like tissue, which encompassed intense extracellular matrix, abundant vasculature, and the deposition of predentin-like tissue equipped with a layer of odontoblast-like cells [25]. In addition to the aforementioned therapeutic efficacy of exosomes on odontogenesis, accumulating evidence has shown that exosomes or EVs secreted by DPSCs can activate diverse cellular processes in MSCs such as migration, proliferation, and osteogenic differentiation, which in turn culminated in promoted bone regeneration [26], [27], [28]. Collectively, the feasibility of exosomes in fostering odonto/osteogenesis brings a novel perspective for developing exosomes as promising nanotherapeutic agents for pulp-dentin complex regeneration and raises the question of how to acquire exosomes with more enhanced therapeutic effects. From a developmental viewpoint, we envisioned that DPSC-Od-Exos mimicking the odontogenesis microenvironment would elicit a more robust potential than DPSC-Exos in promoting DPSC odontogenic differentiation. To elucidate this hypothesis, we delineated that both DPSC-Od-Exos and DPSC-Exos could be substantially internalized by DPSCs, displaying a pronounced stimulatory effect on DPSC proliferation. In particular, as compared to DPSC-Exos, DPSC-Od-Exos not only considerably enhanced the migration capacity of DPSCs within 24h but also remarkably facilitated their odontogenic differentiation, resulting in the upregulation of odonto/osteogenic-related gene expression, increase in ALP activity, and promotion of mineral deposition. Therefore, DPSC-Od-Exos were superior to DPSC-Exos in orchestrating DPSC fate and ameliorating their odontogenic differentiation capability. Mechanistically, this superiority can be attributed to the component alteration in exosomes originating from DPSCs that have undergone odontogenic differentiation during the initial phase. In line with our findings, a prior investigation illuminated that DPSC-Od-Exos harvested under odontogenic conditions for 4 weeks performed better than regular DPSC-Exos in triggering the upregulated expression of genes essential for odontogenesis, which consequently yielded a more robust expression of odontogenic proteins following subcutaneous implantation in mouse models [20]. This emphasized the assertion that DPSC-Od-Exos were more appropriate to induce DPSC odontogenic differentiation compare with DPSC-Exos. However, given that DPSC odontogenically induced for 4 weeks had ultimately differentiated into mature odontoblast-like cells with mineralizing competence, DPSC-Od-Exos in the above study were more accurately denominated as odontoblast-derived exosomes, which incorporated characteristic biological cues of odontoblasts pivotal for dentinogenesis. Undoubtedly, in our study, the induction of an odontogenic microenvironment has been implicated as a critical contributing factor in optimizing the pro-odontogenic commitment of DPSC-derived exosomes.

Pulp-dentin complex regeneration involves multifaceted and synchronized biological processes. In addition to dentin regeneration, angiogenesis is considered as a preliminary and prerequisite event for functional pulp regeneration, thus supplying vital nutrients and excreting metabolic wastes. In this regard, exosomes have provided a new choice to facilitate pulp angiogenesis. As recently demonstrated by Li et al. (2023) in vitro and in vivo, exosomes derived from stem cells from apical papilla (SCAP) under hypoxic conditions led to a significant enhancement in HUVEC angiogenesis by transferring miRNA-126, which consequently suppressed SPRED1 and triggered ERK signaling pathway [29]. Moreover, concentrated on stem cells from human exfoliated deciduous teeth (SHED), Wu et al. (2021) revealed that SHED aggregate-derived exosomes could promote endothelial cell differentiation of SHED and augment their angiogenic competence through the delivery of miRNA-26a via the regulation of TGF-β/SMAD2/3 pathway [15]. Hence, it has indicated that exosomes hold immense potential in pulp revascularization not only via a direct proangiogenic effect on HUVECs but also through indirectly improved endothelial differentiation of stem cells. Intriguingly, in the present study, we have confirmed that DPSC-Exos at 50 μg/mL possessed extraordinary capacity to foster angiogenesis in HUVECs and DPSCs at both cytological and histological levels, which was in accordance with previously published reports [21], [30]. However, it was worth emphasizing that compared with DPSC-Exos, DPSC-Od-Exos exhibited superior proliferative, migratory, and proangiogenic effects on HUVECs and DPSCs in vitro. More importantly, in vivo compelling evidence from Matrigel plug assays and human tooth root fragment transplantation collectively established that DPSC-Od-Exos showed a remarkable promoting role in the formation of enriched vascular structures. Considering that DPSCs have broader tissue sources and are much easier and more convenient to obtain than SCAP and SHED, DPSC-Od-Exos present exciting opportunities for future advancements in pulp angiogenesis.

As a highly innervated tissue, dental pulp contains abundant neural networks originating from the trigeminal ganglion and cervical sympathetic ganglion, which constitute 40% of dental pulp volume [31], [32]. Dental nerves play crucial roles in sensory conduction and take responsibility for the regulation of pulpal blood flow. In addition, 20% of pulp vasculature has been unveiled to display distinctive interactions with pulp neural networks, substantially highlighting the intimate association between neural architecture and blood vessels in dental pulp [32]. Accordingly, in the context of regenerative endodontics, the regeneration of neural structures is indispensable for achieving functional recovery of neurovascular system in dental pulp. Recently, various non-dental exosomes have been extensively exploited as promising therapeutic tools to stimulate repair and regeneration of central and peripheral nerve tissues [33], [34], [35]. In particular, aimed at facilitating nerve regeneration and restoring locomotor function in diabetic patients with peripheral nerve injury (PNI), Yang et al. (2023) illustrated that bone marrow MSC-derived exosomes encapsulated in hydrogels not only attenuated inflammatory pain by triggering M2 macrophage polarization but also propelled myelinated axonal outgrowth in rats after diabetic PNI, thus alleviating muscular denervation atrophy and ameliorating motor function [36]. Besides the aforementioned PNI, an accumulating body of evidence has suggested that exosomes confer favorable neurotherapeutic benefits by inhibiting neuronal apoptosis and inflammation while boosting axonal regeneration, consequently contributing to the repair of traumatic brain injury and spinal cord injury [37], [38], [39]. Given that DPSCs are cranial neural crest-derived cells, exosomes secreted by DPSCs are expected to provide more prominent advantages in neuroregeneration and neuroprotection in comparison to exosomes obtained from other cell sources. According to Chai et al. (2024), the administration of DPSC-Exos could considerably suppress the autophagy of Schwann cells through shuttling miRNA-122-5p via the inhibition of P53 pathway, which thereby supported myelin sheath regeneration in rats following sciatic nerve injury [40]. In line with this, it was well validated by Liu et al. (2022) that DPSC-Exos with the capability of diminishing M1 macrophage transition markedly mitigated inflammatory process and attenuated neurological impairments in spinal cord-injured mice by impeding ROS-MAPK-NFκB P65 signaling [41]. Together, these findings shed light on the solid neuroregenerative and neuroprotective potential of DPSC-Exos in addressing diverse neurological disorders. Notably, the specific efficacy of DPSC-Exos in promoting pulp neurogenesis has garnered scant research attention. At this point, DPSC-Exos in our study have been verified to exhibit a promoting effect on neuronal marker expression at both mRNA and protein levels. Of importance, in sharp contrast with DPSC-Exos, DPSC-Od-Exos elicited a remarkable enhancement in the expression of *GDNF* and *Nestin* in vitro, as well as the formation of neural networks positively stained by NF200 in vivo. These findings indicate the robust neurotrophic competence of DPSC-Od-Exos in boosting pulp innervation recovery and regeneration. To the best of our knowledge, the present study illuminates for the first time that DPSC-Od-Exos represent a novel and prospective therapeutic modality for pulp neuralization. Hence, DPSC-Od-Exos have emerged as a conceivable strategy to achieve complete functional pulp-dentin complex regeneration, which is critical for sustaining tooth vitality. Furthermore, our results give valuable insights into the promising application of DPSC-Od-Exos for addressing both central and peripheral nerve disorders. In the future, we will dedicate intense efforts to uncovering the detailed molecular mechanism by which DPSC-Od-Exos exert desirable neurogenic effectiveness.

Collectively, by recapitulating the developmental microenvironment, DPSC-Od-Exos possess multifaceted capacity for pulp-dentin complex regeneration through the delivery of pivotal bioactive molecules, which synergistically facilitate tubular dentin formation and enhance neurovascular structure reconstruction in mouse models. However, several limitations should be underscored in the current study. First, the regulatory mechanisms through which DPSC-Od-Exos operate remain elusive. Regarding exosomes encompass a variety of biological substances, their capacity for intercellular communication suggests an intricate network of regulatory interactions in recipient cells. Implementing comprehensive transcriptomic, proteomic, and metabolomic analyses would elucidate the crucial biomolecules and delineate decisive signaling and metabolic pathways driving certain biological process of DPSC-Od-Exos. In particular, miRNAs, which represente the most copious RNA content in MSC-derived exosomes [42], have been identified to participate in the regulation of diverse biological processes. miRNAs are endogenous, small, single-stranded, non-coding RNA molecules, typically comprising 19–24 nucleotides in length. In addition to the aforementioned exosomal miRNA-mediated intercellular communications during pulp-dentin complex regeneration, accumulating research has revealed that specific miRNAs transferred by exosomes or EVs, such as miRNA-758-5p and miRNA-27b-5, displayed an outstanding modulating effects on the expression of critical markers associated with odontogenic differentiation, including ALP, DSPP, RUNX-2, and osterix [43], [44]. Furthermore, several miRNAs, including miRNA-124-3p, miRNA-199-5p, and miRNA-214, have demonstrated the capability to promote M2 macrophage polarization, Schwann cell proliferation and migration, as well as axonal myelination and extension, strongly implying their desirable potential to facilitate neurogenesis and neuroprotection [35], [45], [46]. Besides, the enrichment of miRNA-31 and miRNA-21 has been implicated in proangiogenic activities, which enhanced endothelial differentiation and supported vascular regeneration [47], [48]. In light of these findings, considerable attention should be directed towards RNA sequencing to discover the various regulatory roles of miRNAs in exosome-induced pulp-dentin regeneration. Moreover, it is noteworthy that exosomes also incorporate versatile proangiogenic factors, such as VEGF, PDGF, TGF-β, and MMP-9 [49], [50]. Therefore, it is speculated that exosome-mediated angiogenesis is attributable to the synergistic actions of both miRNAs and peptide constituents. Accordingly, there exists a pressing need to identify novel peptide components that potentiate angiogenesis, odontogenesis, and neurogenesis, which could be achieved through a detailed evaluation of the proteomics profile of exosomes. It is essential to highlight that one of the limitations of our in vivo assays arises from the inability to accurately replicate the hypoxic conditions prevalent in actual root canals through unsealed tooth root fragments, despite the satisfactory regenerative effects associated with DPSC-Od-Exos. From a translational perspective, such manipulation may create an artificially more conducive environment for tissue regeneration than what is typically encountered in the dental pulp, potentially constraining the applicability of our findings. To mitigate this limitation, further elucidation is required regarding how variations in sealing impact study outcomes, emphasizing the necessity of replicating physiological hypoxic conditions in future investigations. Additionally, orthotopic regeneration of pulp-dentin complex has yet to be validated in large animal models, such as dogs and minipigs. To address this concern, the combinatorial employment of small and large animal models is considered crucial for providing more convincing evidence to enhance the therapeutic prospects of DPSC-Od-Exos in clinical settings. Concurrently, in an attempt to obtain more accurate regeneration details during animal studies, establishing a continuous observation framework across different time points warrants significant scientific merit and should be prioritized in the forthcoming preclinical research involving large animal models.

## Conclusion

In summary, this study presents a remarkable advancement in the field of regenerative endodontics by illustrating, for the first time, the extraction of DPSC-Od-Exos from DPSCs that have undergone initial odontogenic differentiation while preserving their MSC characteristics. Encouragingly, DPSC-Od-Exos exhibit tremendous prospects for developing as a promising therapeutic strategy, taking multifaceted roles in promoting the complete regeneration of pulp-dentin complex. As investigated in vitro, DPSC-Od-Exos were capable of optimizing the biological functions of DPSCs and HUVECs, facilitating their proliferation and migration. In striking contrast with normal DPSC-Exos, DPSC-Od-Exos contributed to a significant enhancement in DPSC odontogenic and neurogenic differentiation in vitro, which was further verified by newly formed tubular dentin layer accompanied by odontoblast-like cells extending cytoplasmic processes and abundant neural structures distributed in pulp tissue at the histological level. In addition, DPSC-Od-Exos were superior to DPSC-Exos in stimulating DPSC endothelial differentiation and HUVEC angiogenesis, thereby posing positive implications to the reconstruction of dense vascular networks with relatively expanded lumens within pulpal space. Hence, by simulating the developmental microenvironment, multifunctional DPSC-Od-Exos expand our knowledge of their therapeutic efficacy in regenerative endodontic practice. Notably, due to the limitations of the present study, the intricate molecular mechanisms underlying the effectiveness of DPSC-Od-Exos warrant more profound research in the future. Moreover, in situ orthotopic pulp regeneration using large animal models deserves to be explored. To sum up, our findings compensate for the restrictions of conventional growth factor administration and lay a solid foundation for further advancements in the field of regenerative endodontics. Representing novel nanotherapeutic tools, DPSC-Od-Exos show enormous potential to achieve structural regeneration and functional reconstruction of pulp-dentin complex.

## Funding

This work was supported in part by National Natural Science Foundation of China (No. 82401074), Natural Science Foundation of Hubei Province (No. 2022CFB717), Youth Fund of Chinese Stomatological Association (No. CSA-MWO2021-03), Undergraduate Teaching Project of Huazhong University of Science and Technology (No. 2022201), Undergraduate Innovation and Entrepreneurship Training Program of Hubei Province (No. S202410487179), General Project of Teaching Research Fund of Second Clinical College, Tongji Medical College, Huazhong University of Science and Technology (No. 2022058), and Cultivation Project of Scientific Research Fund of Tongji Hospital, Tongji Medical College, Huazhong University of Science and Technology (No. 2022B25). Meanwhile, we sincerely appreciate the technical assistance from the Experimental Medicine Research Center, Tongji Hospital, Tongji Medical College, Huazhong University of Science and Technology during experiment implementation.

## Declaration of competing interest

The authors declare that they have no known competing financial interests or personal relationships that could have appeared to influence the work reported in this paper.
