# Supplementary material for: Odontogenic exosomes simulating the developmental microenvironment promote complete regeneration of pulp-dentin complex in vivo
Source: J Adv Res. 2025 Jan 5;76:405–21. doi: 10.1016/j.jare.2024.12.048 (PMC12793753; doi:10.1016/j.jare.2024.12.048)
Supplement: Supplementary Data 1 [file mmc1.zip › Figure S1 Exosomes facilitated the proliferation of DPSCs and HUVECs in a time- and dose-dependent manner.pdf]

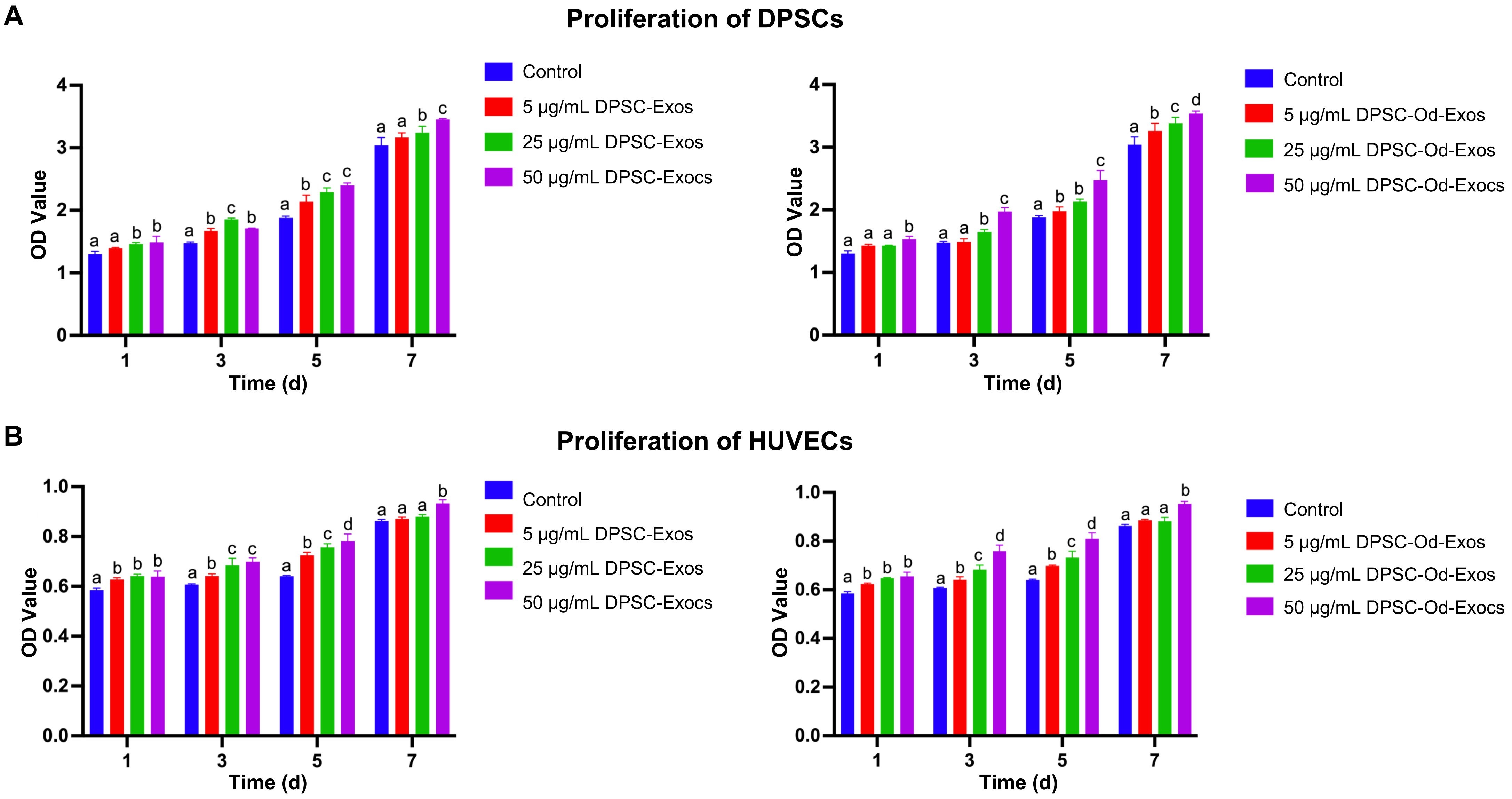

Figure S1 Exosomes facilitated the proliferation of DPSCs and HUVECs in a time- and dose-dependent manner. Both DPSC-Exos and DPSC-Od-Exos could time- and dose-dependently enhance the proliferative capability of DPSCs (A) and HUVECs (B). Different letters above the bars indicated statistically significant difference at $p < 0.05$.
