# Supplementary material for: Odontogenic exosomes simulating the developmental microenvironment promote complete regeneration of pulp-dentin complex in vivo
Source: J Adv Res. 2025 Jan 5;76:405–21. doi: 10.1016/j.jare.2024.12.048 (PMC12793753; doi:10.1016/j.jare.2024.12.048)
Supplement: Supplementary Data 1 [file mmc1.zip › Table S1 RT-qPCR Primer Sequences.pdf]

Table S1. RT-qPCR Primer Sequences

Gene	Primer sequences (5' - 3')
Human β-actin	F: TGGCACCCAGCACAATGAA
	R: CTAAGTCATAGTCCGCCTAGAAGCA
ALP	F: ATGGGATGGGTGTCTCCACA
	R: CCACGAAGGGGAACTTGTC
ANG II	F: CTGGGCGTTTTGTTGTTGGTC
	R: GGTTTGGCATCATAGTGCTGG
BMP-2	F: CGTCAAGCCAAACACAAACAG
	R: GCCACAATCCAGTCATTCCAC
Col1α	F: GATTCCCTGGACCTAAAGGTG
	R: AGCCTCTCCATCTTTGCCAGCA
DMP-1	F: TACAGCATGTCCTACTCGCAG
	R: GAGGAAGAGGTAACCACAGGG
OCN	F: GGCGCTACCTGTATCAATGG
	R: GTGGTCAGCCAACTCGTCA
RUNX-2	F: TGGTTACTGTCATGGCGGGTA
	R: TCTCAGATCGTTGAACCTTGCTA
MMP-9	F: TGTACCGCTATGGTTACACTCG
	R: GGCAGGGACAGTTGCTTCT
VEGF	F: AGGGCAGAATCATCACGAAGT
	R: AGGGTCTCGATTGGATGGCA
PDGFA	F: GCAAGACCAGGACGGTCATTT
	R: GGCACTTGACACTGCTCGT
SOX2	F: TACAACTCCATGACCAGC
	R: CTTGACCACCGAACCCAT
DSPP	F: TGGCGATGCAGGTCACAAT
	R: CCATTCCCACTAGGACTCCCA
Nestin	F: GAAGGGCAATCACAACAGGTG
	R: GGGGCCACATCATCTTCCA
GDNF	F: GCAGACCCATCGCCTTTGAT
	R: CCACACCTTTTAGCGGAATGC
