# Supplementary figures and images for: Odontogenic exosomes simulating the developmental microenvironment promote complete regeneration of pulp-dentin complex in vivo
Source: J Adv Res. 2025 Jan 5;76:405–21. doi: 10.1016/j.jare.2024.12.048 (PMC12793753; doi:10.1016/j.jare.2024.12.048)

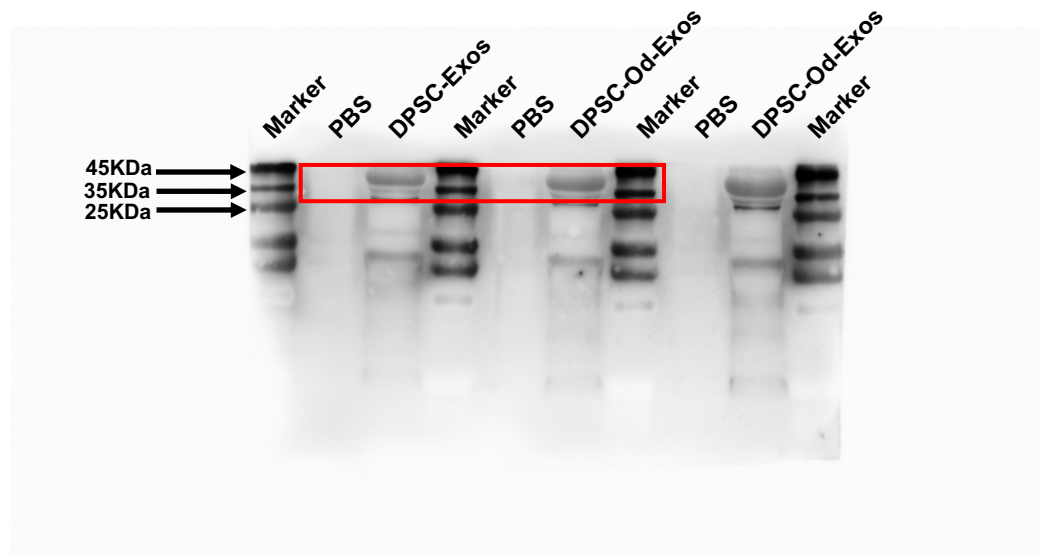

TSG101: 44KDa

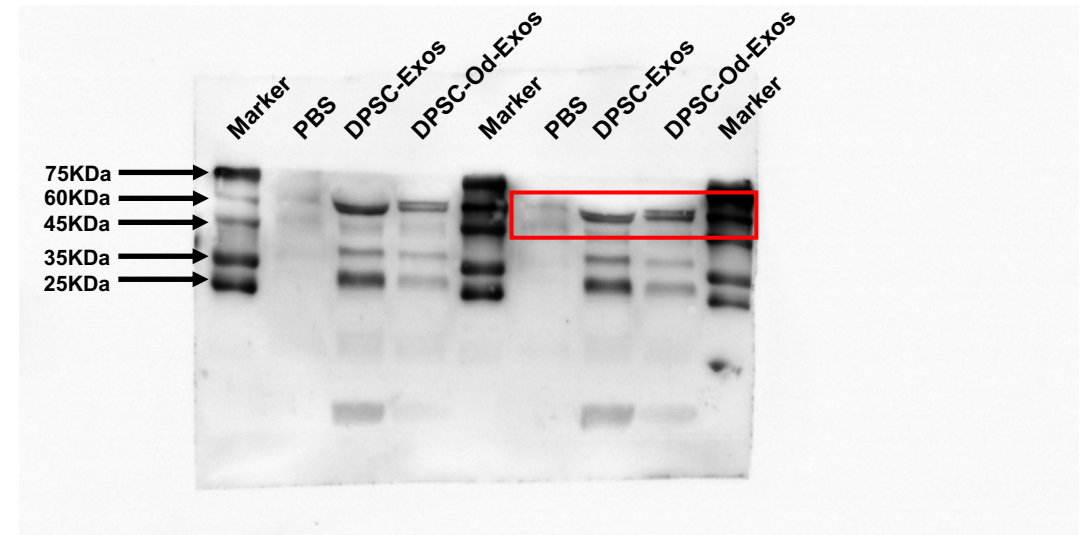

CD63: 53KDa

Supplement: Supplementary Data 1 [file mmc1.zip › Original WB.pdf]
